# Two Isoforms of the Guanine Nucleotide Exchange Factor, Daple/CCDC88C Cooperate as Tumor Suppressors

**DOI:** 10.1038/s41598-019-48420-w

**Published:** 2019-08-20

**Authors:** Jason Ear, Ying Dunkel, Yash Mittal, Blaze B. C. Lim, Lawrence Liu, Magda K. Holda, Ulrich Nitsche, Jorge Barbazán, Ajay Goel, Klaus-Peter Janssen, Nicolas Aznar, Pradipta Ghosh

**Affiliations:** 1 Department of Medicine, University of California, San Diego, La Jolla, California USA; 2 Department of Cellular and Molecular Medicine, University of California, San Diego, La Jolla, California USA; 3 Department of Surgery, Klinikumrechts der Isar, TechnischeUniversitätMünchen, Munich, Germany; 4 Translational Medical Oncology Laboratory, Health Research Institute of Santiago (IDIS), SERGAS., Santiago de Compostela, Spain; 5 Division of Gastroenterology, Department of Internal Medicine and Charles A. Sammons Cancer Center and Baylor Research Institute, Baylor University Medical Center, Dallas, Texas USA; 6 Cancer Research Center of Lyon, Centre Léon Bérard, Lyon, France; 7 Moores Cancer Center, University of California, San Diego, La Jolla, California USA

**Keywords:** Colon cancer, Cell signalling

## Abstract

Previously, Aznar *et al*., showed that Daple/CCDC88C enables Wnt receptors to transactivate trimeric G-proteins during non-canonical Wnt signaling via a novel G-protein binding and activating (GBA) motif. By doing so, Daple serves two opposing roles; earlier during oncogenesis it suppresses neoplastic transformation and tumor growth, but later it triggers epithelial-to-mesenchymal-transition (EMT). We have identified and characterized two isoforms of the human Daple gene. While both isoforms cooperatively suppress tumor growth via their GBA motif, only the full-length transcript triggers EMT and invasion. Both isoforms are suppressed during colon cancer progression, and their reduced expression carries additive prognostic significance. These findings provide insights into the opposing roles of Daple during cancer progression and define the G-protein regulatory GBA motif as one of the minimal modules essential for Daple’s role as a tumor suppressor.

## Introduction

We previously defined a novel paradigm in Wnt signaling in which Wnt receptors, Frizzled (FZDs), activate G-proteins and trigger non-canonical Wnt signaling via Daple/CCDC88C^1^. Daple, a multimodular signal transducer and a cytosolic protein, was originally discovered as a Dishevelled (Dvl)-binding protein that regulates Wnt signaling^2,3^. Subsequent work showed that Daple directly binds ligand-activated FZDs, and serves as a guanine-nucleotide exchange factor (GEF) that activates the G-protein, Gαi^1^. Binding to the G-protein is mediated via Daple’s C-terminally located Gα-binding and activating (GBA) motif; such binding triggers non-canonical activation of Gαi^1^. Binding to FZD is also brought on via a C-terminally located stretch distal to the GBA motif. Upon ligand stimulation, Daple dissociates from Dvl, binds to and displaces Dvl from FZDs, and assembles a Daple:Gαi complexes on the receptor. How Daple:Dvl complexes are disassembled was unknown until recently, when we demonstrated that phosphorylation of Daple’s PDZ-binding motif (PBM) by both receptor and non-receptor tyrosine kinases can trigger this change^4^. Disassembly of Daple:Dvl complexes and formation of FZD:Daple:Gαi complexes facilitates the activation of Gαi near ligand-activated Wnt receptors. Daple activates Gαi within the FZD:Daple:Gαi complexes; such non-canonical activation of Gαi by Daple-GBA suppresses cAMP, whereas released ‘free’ Gβγ heterodimers enhance Rac1 and PI3K-Akt signals^1^. Although Daple-dependent enhancement of non-canonical Wnt signals can suppress tumor growth^1^, it can also fuel EMT, trigger cancer cell migration and invasion^1,5^ and drive metastasis^6^. Furthermore, elevated expression of Daple-GBA in circulating tumor cells prognosticates a poor outcome^7^. In doing so, Daple serves a dual role during oncogenic progression—a tumor suppressor early during oncogenesis and driver for metastatic invasion later.

Here we identified a novel Daple isoform (Daple-V2) which corresponds only to the C-terminal region of full length Daple (Daple-fl; RefSeq NM_001080414). We demonstrate that Daple-V2 possesses all the functional domains to bind Dvl, FZD7 and Gαi and represents the smallest autonomous Daple unit able to inhibit the β-catenin/TCF/LEF pathway and suppress tumor cell growth. We also demonstrate that both isoforms have different subcellular distribution, and unlike Daple-fl, Daple-V2 does not enhance tumor cell invasiveness and may, therefore, serve as a more effective tumor suppressor and a better prognostic marker than Daple-fl.

## Results and Discussion

### Identification of various isoforms of Daple in cells

We noted that there are 6 other transcript variants catalogued in Ensembl and/or UniProt databases: V2 (552aa), V3 (506aa), V4 (478aa), V5 (96aa), V6 (88aa) and V7 (234aa) (Supplementary Fig. 1A). Among them, V2, V3 and V4 are predicted to encode stretches within Daple’s C-terminus, whereas V5, V6 and V7 are predicted to encode stretches within Daple’s N-terminus. Because distinct protein isoforms generated from single genes are known to contribute to the diversity of the proteome^8^, we asked if Daple’s seemingly opposite and bi-faceted roles in cancers may be due, in part, to the functions of varying isoforms of the same protein. Because prior work by others and us have demonstrated the importance of the PBM and GBA modules within the C-terminus of Daple in the regulation of Wnt signaling, we narrowed our analysis to transcripts encoding Daple’s C-terminus.

**Figure 1:**
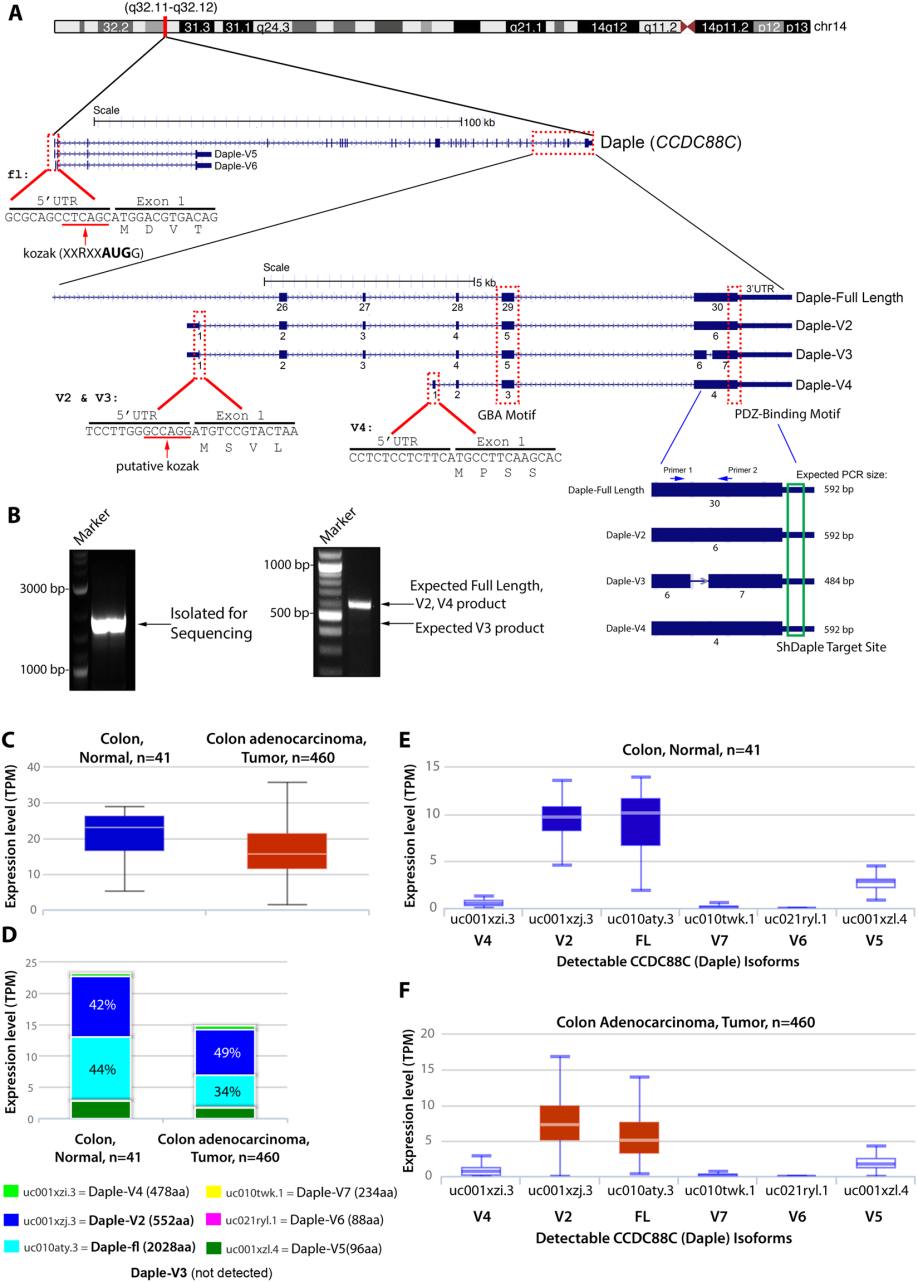
Identification of a novel short isoform of CCDC88C (Daple-V2). (**A**) Schematic showing the various N-terminal and C-terminal transcripts of Daple. Various isoforms containing the unique modular C-terminus of the protein are highlighted. All C-terminal transcripts contain an exon coding a GBA motif and a PDZ-Binding motif. Daple-V2 and Daple-V3 transcripts contain a unique 5′ end that is transcribed from an intronic region between exon 25 and 26 of the full-length gene. This 5′ end contains exon 1 of Daple-V2 and Daple-V3 and codes for a unique N-terminal peptide on these isoforms. The 5′ UTR of the two isoform contains a putative kozak sequence. Daple-V4 contains a unique 5′ end that is transcribed from an intronic region between exon 27 and 28 of the full-length gene. Names of the RNA transcript and encoded protein is indicated on the right. A common 3′ UTR, that is targeted using ShRNA, is located on all transcripts containing the GBA and PDZ-Binding motif. Primers used to detect missing sequence on Daple-V3 transcript is indicated. **(B)** *Left*, PCR from HeLa cDNA using primers to entire coding sequence of Daple-V2/V3. Band was isolated into a cloning vector and sequenced. *Right*, PCR from HeLa cDNA using primers flanking deleted region on Daple-V2, V3 and V4 (corresponding to exon 30 of Daple-fl). **(C–F)** The expression level of various Daple (CCDC88C) transcripts in normal colon and colorectal cancer tissues annotated in The Cancer Genome Atlas (TCGA) Data Portal analyzed by ISOexpresso, a web-based platform (see *Methods*; http://wiki.tgilab.org/ISOexpresso/). Box plots (**C**) display the levels of expression of Daple in normal colon vs. tumor tissues expressed as TPM, Median transcripts per million; TPM was calculated by multiplying the median value of the estimates of the transcript in each sample group by one million. Bar graphs (**D**) display the relative abundance of each Daple transcript in normal vs tumor tissues. Box plots display the absolute abundance of each Daple transcript in normal colon (**E**) and colorectal tumor (**F**) tissues.

We noted that Daple-V2 and V3 differs from Daple-fl by a unique 5′ end **(**Fig. 1A**)** which adds a sequence comprised of 5 amino acids (MSVLS) on the N-terminus of the isoforms. Upon inspection of the V2 and V3 variant, we noticed that both transcripts contained the same transcript ID but are different version of the same transcript (Supplementary Fig. 1A). This arose from annotation of various assemblies in the Ensembl database (V2 from assembly GRCh37 and V3 from GRCh38). In order to confirm if one or both transcripts exist in the cell, we utilized a common primer set to amplify both the V2 and V3 gene from cDNA. Surprisingly only one amplicon was seen from the PCR analysis (Fig. 1B). This product was isolated and cloned into an expression vector and transfected into cells for further analysis.

The V3 transcript is unique from the other C-terminus transcript in that it is missing a stretch of amino acid in exon 30 (using Daple-fl nomenclature, Fig. 1A). To further confirm if, between V2 and V3, V2 is the sole species, PCR analysis on a region flanking the missing sequence was performed (Fig. 1B). No product corresponding to V3 was observed, indicating that Daple-V2 is the major, and perhaps the only transcript between V2 and V3.

Analysis of the 5′-untranslated region (UTR) between Daple-fl, V2, and V4 revealed that the full-length transcript contains a putative consensus kozak sequence (high strength of prediction), with two notable features – a G on the +4 and an A on the −3^9,10^. The putative kozak sequence on V2 only has one of these features (an A on the −3 end), while V4 has neither property on its 5′UTR (Fig. 1A). In summary, when ranked by kozak strength Daple-fl was predicted to be highest, followed by Daple-V2 and then Daple-V4. This finding suggests that V2 might have a higher translation efficiency and thus may translate into a protein that is highly abundant. When we assessed the expression of these C-terminal isoforms, we found that both V2 and V4 transcripts were detected in cultured cells and colon tissues (Supplementary Fig. 2).

### Daple-V2 is the most abundant isoform that is expressed in both normal and tumor tissues

To determine how the expression of Daple isoforms change during normal to cancer progression, we analyzed the relative transcript expression of each Daple isoform in normal and tumor tissues annotated in the TCGA (The Cancer Genome Atlas) data portal using the web server- ISOexpresso. This server facilitates expression-based isoform-level analysis. Such analysis revealed that the cumulative levels of Daple transcripts are reduced from a median of 23.14 to 15.6 between normal colon and colorectal tumor tissues, respectively (Fig. 1C), however, Daple-V2 remained the most abundant isoform (besides Daple-fl) in both cases (Fig. 1D–F).

When we expanded this analysis to include all 9,690 tumors and 732 normal controls of 30 cancer types annotated in the TCGA data portal, Daple-V2 still remained the most dominant isoform across all tissue types (Supplementary Fig. 3A,B). These findings indicate that Daple-V2 is the most abundant isoform of Daple (in normal and tumor tissues) that is translated into protein and has the minimal motifs/modules necessary to scaffold Dvl, FZD7 and G-proteins.

### Validation of Daple-V2, a major isoform of Daple in cells

**Figure 2:**
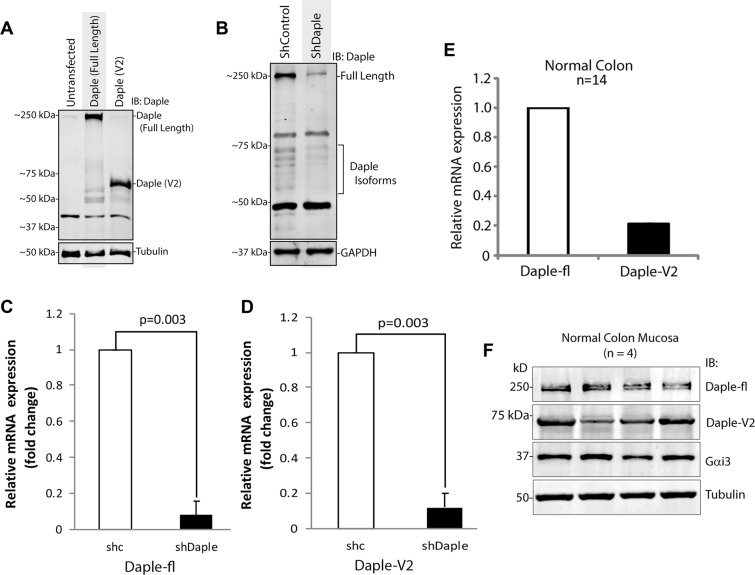
Validation of Daple-V2 in cell lines and human colon tissues. (**A**) Whole cell lysates of HeLa cells transfected with Daple-fl or Daple-V2 were analyzed for expression by immunoblotting for Daple and tubulin. **(B)** Lysates of HeLa cells stably expressing control shRNA (shControl) or shRNA to the 3′UTR on Daple (shDaple) were analyzed for Daple expression by immunoblotting. **(C**,**D)** mRNA isolated from shControl (shC) or shDaple cells were analyzed for Daple-fl (**C**) or V2 (**F**) levels by qPCR. Transcript levels were analyzed relative to shControl. **(E)** mRNA isolated from 14 normal colon samples were analyzed for the expression of full length or V2 isoform of Daple. Relative mRNA expression of Daple-fl and Daple-V2 isoforms is displayed as bar graphs which represent the mean value of 14 samples. **(F)** Whole cell lysates of colonic epithelial from normal subjects were analyzed for Daple, Gαi3 and tubulin by immunoblotting (IB). Both full length (fl) and short isoform (V2) were detected.

We first began by asking if the isolated Daple-V2 gene product that we cloned, actually expressed as a protein. We found that is indeed the case; a product of predicted molecular weight was detected by immunoblotting in cells exogenously expressing the Daple-V2 construct using an antibody that was raised against Daple’s C-terminus (Fig. 2A). Next we asked if Daple-V2 is expressed in cells (endogenous pool). Because all transcripts sharing the C-terminal GBA and PDZ-binding motifs share an identical 3′UTR (Fig. 1A), we utilized a previously-validated 3′UTR-targeted shRNA approach to knockdown various isoforms of Daple in cells^1^. Full length immunoblots revealed that Daple-fl (~250 kDa) and several bands around the corresponding molecular weight of Daple-V2 (~70 kDa) are depleted in shDaple cells compared to shControl (Fig. 2B). Because most of the bands observed ~70 kDa were depleted in shDaple cells, these may represent Daple-V2, its various posttranslational modified forms, and/or other transcript variants. Successful targeting of Daple-fl and Daple-V2 was further confirmed through measurements of mRNA transcripts by quantitative RT-PCR (qPCR). Compared to shControl cells, shDaple cells had a significant decrease in both full length and V2 isoforms (Fig. 2C,D).

Next, we checked the expression of mRNA and protein of Daple-fl and Daple-V2 in normal adult colon. We analyzed mRNA levels of both Daple-fl and Daple-V2 in 14 colons samples by qPCR; both isoforms were detected (Fig. 2E**)**. Furthermore, we confirmed the protein expression of Daple-fl and Daple-V2 (Fig. 2F) by analyzing lysates of mucosal biopsies taken from normal colons by immunoblotting using an anti-Daple-CT antibody raised against aa 1660–2028 ([previously validated in^1^ and expected to detect both Daple-fl and -V2 isoforms).

### Daple-V2 represents the smallest autonomous Daple unit that can bind Dvl, FZD7 and Gαi

**Figure 3:**
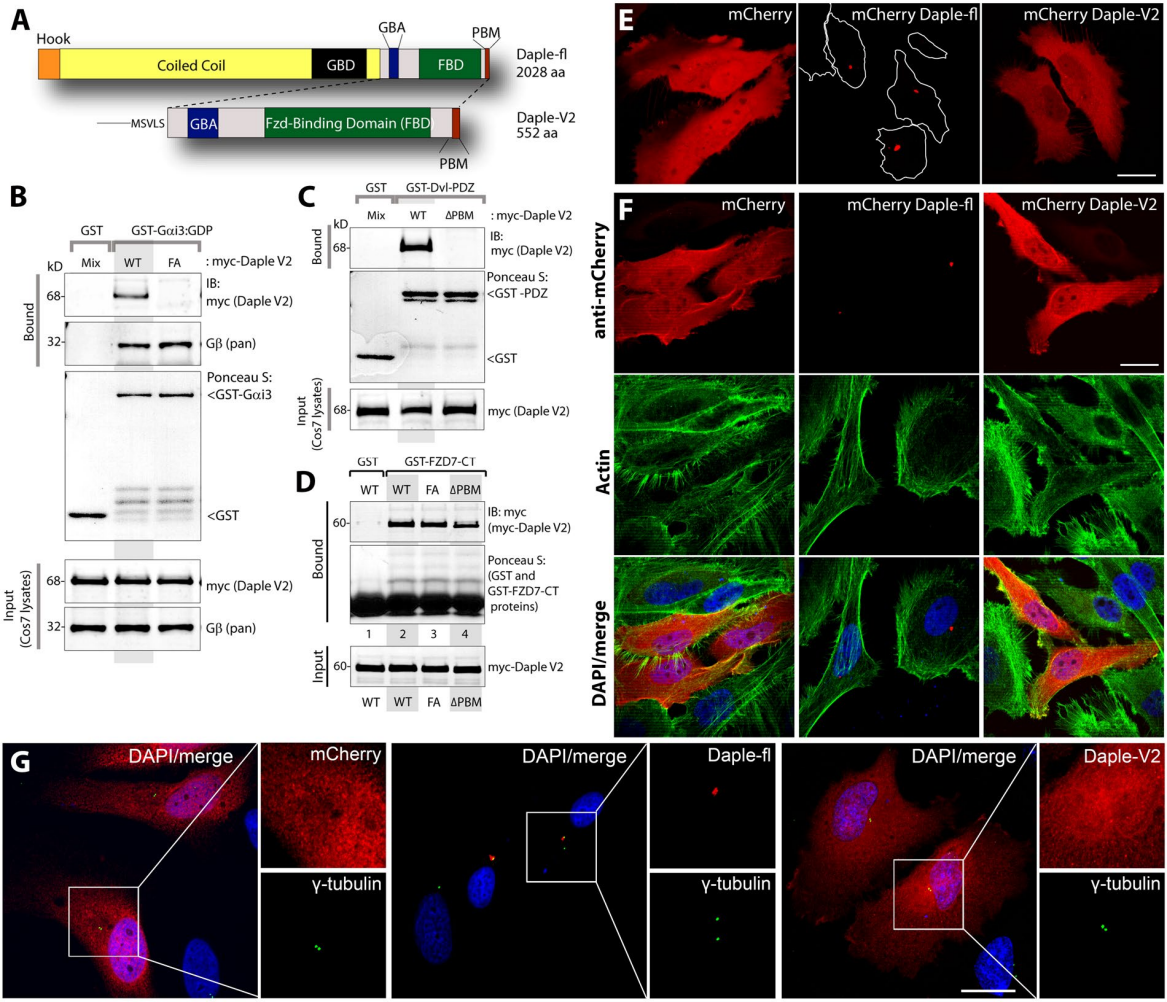
Daple-V2 contains functional C-terminal modules which enable binding to trimeric G-protein subunit, Gαi, Dvl and Frizzled receptor and has a different subcellular localization to Daple-fl. (**A)** Schematic comparing the domain distribution of Daple-fl and the shorter isoform Daple-V2. **(B)** Purified, recombinant GST-Gαi3 preloaded with GDP and immobilized on glutathione-agarose beads was incubated with cell lysates of Cos7 cells (input) expressing myc-Daple-V2 WT or F194A (FA) as indicated. Bound proteins were analyzed for Daple-V2 (myc) and Gβ by immunoblotting (IB). Equal loading of GST-tagged proteins were confirmed by Ponceau S staining. F194A mutation disrupts binding of Daple-V2 to Gαi3. **(C)** Purified, recombinant GST-tagged PDZ domain of Dvl immobilized on glutathione-sepharose beads was incubated with cell lysates of Cos7 cells (input) expressing myc-Daple-V2 WT or delta PBM (ΔPBM) as indicated. Bound proteins were analyzed for Daple-V2 (myc) by immunoblotting (IB). Equal loading of GST-tagged proteins was confirmed by Ponceau S staining. Deletion of the C-terminal PDZ-binding motif disrupts binding of Daple-V2 to PDZ domain of Dvl. **(D)** Purified, recombinant GST-tagged carboxyl terminus of FZD7R (FZD7-CT) immobilized on glutathione-sepharose beads was incubated with cell lysates of Cos7 cells (input) expressing myc-Daple-V2 WT, FA or delta PBM (ΔPBM) as indicated. Bound proteins were analyzed for Daple-V2 (myc) by immunoblotting (IB). Equal loading of GST-tagged proteins was confirmed by Ponceau S staining. WT and mutants of Daple-V2 bound similarly to FZD7R. **(E)** mCherry-tagged Daple-fl, V2, or fluorescent protein alone was transfected into HeLa cells and subjected to live imaging. Scale bar represents 25 μm. **(F)** HeLa cells transfected with mCherryDaple construct, as in (**E)**, were immunostained for mCherry and phalloidin to stain actin structures. Scale bar represent 25 μm. **(G)** HeLa cells transfected with mCherryDaple construct, as in (**E)**, were immunostained for mCherry and γ-tubulin. Scale bar represent 25 μm.

We noted that the V2 isoform possesses the C-terminal region of the full length Daple protein and hence, contains a GBA, a frizzled binding domain, and a PDZ Binding motif (PBM) (Fig. 3A). To study the properties of Daple-V2 and compare them with the previously described full-length protein, we cloned the Daple-V2 transcript into a plasmid for mammalian expression as a N-terminally myc-tagged protein, just as we did previously for Daple-fl^1^. We also generated V2 constructs with previously defined specific mutants of Daple that lack the important motifs, GBA [Daple-V2-FA (F194A)] and PBM [Daple-V2- ΔPBM (deleted from 549–552aa)] and used these mutants to further decipher the properties of Daple-V2. As expected, we confirmed by GST pulldown assays that myc-Daple-V2 interacts with Gαi3, PDZ domain of Dvl and the cytoplasmic tail of FZD7 **(**Fig. 3B–D**)**.These findings indicate that Daple-V2 represents the smallest autonomous Daple unit possessing the key biochemical features of the C-terminus of Daple-fl previously described^1^.

### Daple-V2 and Daple-fl have distinct subcellular localizations

Prior work from our group demonstrated the importance of the N-terminal coiled-coil domain (which is absent in Daple-V2) in localizing Daple to the pericentriolar recycling compartment^11^. Therefore, we next asked if the two isoforms of Daple differs in its subcellular localization. As expected, transfection of mCherry tagged Daple-fl into HeLa cells revealed that the full-length protein localizes to a pericentriolar compartment, as determined by their proximity to the centrosomal marker γ-tubulin (Fig. 3G). By contrast, Daple-V2 is distributed ubiquitously throughout the cell and was seen even within the nucleus (Fig. 3E–G). Immunoblotting using mCherry or Daple antibodies confirmed that the exogenously expressed Daple protein is stable and the observed localization is not due to degradation of the protein (Supplementary Fig. 4). Others have noted that localization of Daple can differ depending on the chemical fixative used^12^. We observed the contrasting localizations of Daple-fl and Daple-V2 regardless of whether cells were live or fixed with different fixatives, i.e., paraformaldehyde or methanol (Fig. 3E–G). These results indicate that the observed difference in the localization of Daple’s isoforms is likely to be real. Because the subcellular localization of a signal transducer is a key determinant of the signals it modulates, our findings also suggest that differential localization of Daple-fl and Daple-V2 may translate to different functional roles in the cell, and some of those functions may be dictated by their compartmentalized actions.

### Daple-V2 antagonizes Wnt signaling via the β-Catenin/TCF/LEF pathway, suppresses growth and proliferation but does not trigger EMT or cell invasion

**Figure 4:**
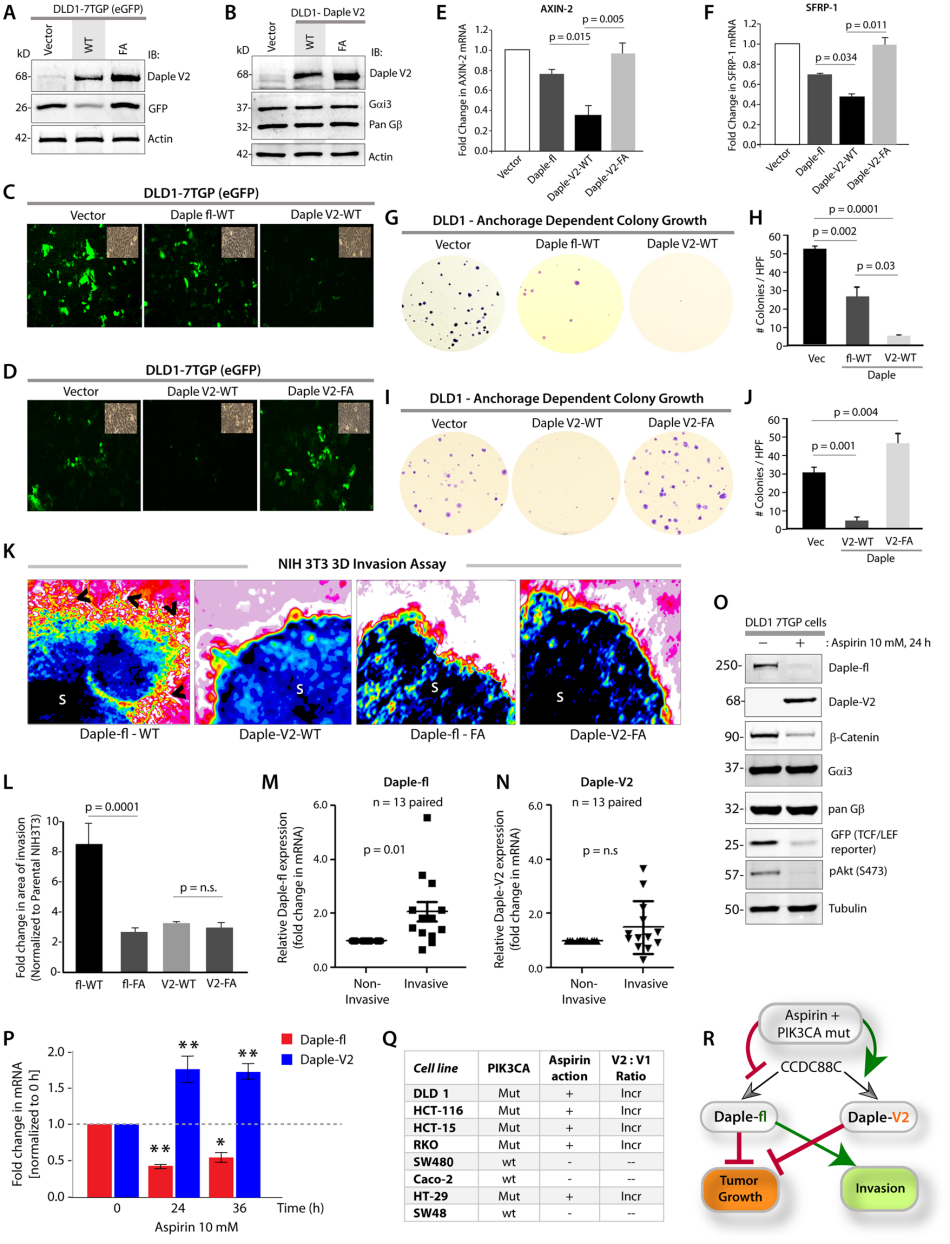
Daple-V2 and -fl have differential effects on tumor cell growth, β-Catenin/TCF/LEF pathway and cell invasion and are differentially expressed in response to Aspirin. (**A)** Whole cell lysates of DLD1 cells stably co-expressing the 7TGP reporter and either vector control or Daple-V2 were analyzed for Daple-V2, GFP and actin by immunoblotting (IB). The intensity of GFP indicates the extent of β-Catenin/TCF/LEF signals. **(B)** Whole cell lysates of DLD1 cells stably expressing vector control, Daple-V2 WT or FA were analyzed for Daple-V2, Gαi3, pan Gβ and actin by immunoblotting (IB). **(C,D)** Monolayers of DLD1 7-TGP cell lines in **A** were starved and stimulated with Wnt5a. Images display representative fields analyzed by fluorescence microscopy. The intensity of eGFP signals denotes Wnt transcriptional activity. Insets show representative fields confirming the confluency of the monolayers in each case. In **C**, compared to DLD1 cells expressing vector control, both Daple-fl-WT and Daple-V2-WT showed inhibition of eGFP; inhibition with Daple-V2 was more robust. In **D**, Daple-V2-WT, but not Daple-V2-FA inhibited eGFP. **(E,F)** HeLa cells transfected with myc-Daple constructs as indicated were analyzed for AXIN-2 and SFRP-1 mRNA by qPCR. Results were normalized internally to mRNA levels of the housekeeping gene, GAPDH. Bar graphs display the fold change in each RNA normalized to the expression in cells expressing control vector. Error bars represent mean ± S.D of 3 independent experiments. **(G–J)** Monolayers of DLD1 cells in (**B)** were analyzed for their ability to form adherent tumor cell colonies on plastic plates during 2–3 weeks prior to fixation and staining with crystal violet. In panels G and I, photographs of representative wells of the crystal violet-stained 6-well plates are displayed. The number of colonies was counted by ImageJ (Colony counter). In panels (H,J**)**, bar graphs display the # of colonies per well seen in each cell line in (**G**,**I)**, respectively. Panels (G,H) show that both Daple-fl and Daple-V2 can inhibit tumor growth. Panels (I,J) show that the GBA motif of Daple-V2 is required for the inhibition of anchorage-dependent colony growth. **(K,L)** Spheroids (S) of NIH3T3 cells expressing WT or FA mutant of myc-Daple-fl or myc-Daple-V2 isoform were analyzed for their ability to invade matrigel in response to Wnt5a (100 ng/ml) using a Cultrex-3D Spheroid Invasion Kit (Trevigen). Representative images of spheroid edges are displayed (**K**). An increase of invasion tracks (arrowheads) was noted only from the edge of tumor spheroids formed by cells expressing myc-Daple-fl-WT, but not the GEF-deficient F1675A (FA) mutant. Neither the WT nor the FA mutant of Daple-V2 could trigger invasion. Area of invasion was quantified using ImageJ and displayed as bar graphs (**L**). Error bars represent mean ± S.D of 3 independent experiments. **(M,N)** Paired samples from non-invasive center and the invasive edges of colorectal cancers were analyzed for Daple-fl (**M**) and Daple-V2 (**N**) expression by qPCR. Scatter plot displays the relative abundance of Daple expression. Daple-fl, but not Daple-V2 is increased in the invading margins of tumors compared to the non-invasive tumor cores. Error bars represent mean ± S.D. n = 13. **(O)**. Immunoblots showing the impact of low dose Aspirin on endogenous Daple isoforms expressed by DLD1-7-TGP cells. β-Catenin and GFP were assessed as positive controls; the abundance of GFP serves as a surrogate marker for the transcriptional activity of β-Catenin via the TCF/LEF axis. G proteins, Gα and β-subunits were assessed as negative controls. **(P)** Parental DLD1 cells were analyzed for Daple-fl and Daple-V2 mRNA by qPCR at indicated time points after exposure to 10 mM Aspirin. Bar graphs display the fold change in each mRNA (Y axis) normalized to the expression levels at 0 hour. Error bars represent mean ± S.D of 3 independent experiments. *p* values: *=<0.05; **<0.01. (**Q**) Table summarizing the various CRC cell lines, their PIK3CA status^50^, their sensitivity to the anti-proliferative action of Aspirin^15^, and the impact of Aspirin on the ratio of Daple-V2:Daple-fl transcripts (see Supplementary Fig. 6). Mut = mutant; + = significant growth suppression when exposed to Aspirin^15^; Incr = increased. (**R**) Schematic summarizing the effect of the recently discovered GBA motif in Daple-fl and Daple-V2 on tumor growth and tumor invasion, and how Aspirin alters the levels of expression of each isoform. The GBA motif of Daple-fl inhibits tumor growth and enhances tumor invasion, whereas the GBA motif of Daple-V2 exclusively inhibits tumor growth. Red lines = Inhibition. Green lines = Enhancement. Thickness of the lines depicts the relative strength of phenotypes.

We previously demonstrated that Daple-fl and more specifically its GBA motif, antagonizes the β-catenin-dependent Wnt signaling pathway and inhibits colony growth, but enhances the PI3K-Akt and Rac1 signals, EMT and invasion^1^. Because Daple-V2 possesses a GBA motif, and because the motif is required for binding G-proteins, we asked if this motif is functional in cells. Using DLD1 colon cancer cells stably expressing 7-TGP **(**Fig. 4A**)**, an eGFP expressing Wnt activity reporter construct^1^, or parental DLD1 cells (Fig. 4B), we generated stable cell lines expressing Daple-V2 wild-type (WT) and GEF-deficient (F194A; FA) mutant. For comparison, we used previously developed and characterized^1^ DLD1 lines expressing the Daple-fl-WT.

We found that Daple-V2 and Daple-fl have two similarities and one dissimilarity. First similarity was that both Daple-fl and Daple-V2 antagonize the β-catenin/TCF/LEF pathway **(**Fig. 4C**)**; Daple-V2-WT, but not Daple-V2-FA, inhibited eGFP expression (Fig. 4A,D), indicating that the inhibitory effect of Daple-V2 on the canonical Wnt pathway required an intact GBA motif. Consistently, both Daple-fl and Daple-V2 reduced the transcription of downstream target genes Axin-2 and SFRP-1; once again, the presence of an intact GBA motif was critical for such inhibition (Fig. 4E,F). Second similarity was that expression of either Daple-fl or Daple-V2 inhibited anchorage-dependent colony growth of DLD1 cells by ~50% and 90%, respectively (Fig. 4G,H**)**. Such growth suppressive effect required an intact GBA motif because, compared to Daple-V2-WT, expression of the GBA-deficient F194A [^1^ corresponding to F1675 in Daple-fl; henceforth, FA] mutant not only failed to inhibit cell colony formation, but also enhanced anchorage-dependent growth **(**Fig. 4I,J**)**.

The dissimilarity between Daple-fl and Daple-V2 was observed in whether they used their GBA motifs to trigger EMT. Compared to cells expressing the GBA-deficient FA mutants of Daple-V2 or Daple-fl, those expressing Daple-fl WT had significantly higher expression of Lox-L3 and Vimentin, two genes commonly associated with epithelial-mesenchymal transition (EMT) **(**Supplementary Fig. 5**)**. Furthermore, in 3-D matrigel invasion assays using the transformed NIH3T3 cells, as done previously^1^, enhanced invasion, as determined by the area of invasion was detected exclusively in the presence of Daple-fl-WT, but not in cells expressing Daple-V2 or Daple-fl-FA, indicating that only Daple-fl can trigger cell invasion (Fig. 4K,L). We found that expression of Daple-fl, but not Daple-V2 is increased in the invading margins of tumors compared to the non-invasive tumor cores **(**Fig. 4M,N**)**, which is in keeping with our findings that Daple-V2 does not contribute to EMT or higher invasiveness.

### The ratio of Daple-fl and V2 isoforms shift when cells are exposed to Aspirin

Because both Daple isoforms can suppress the β-catenin/TCF/LEF pathway and anchorage-dependent colony growth, but only Daple-fl can induce EMT and invasiveness, we rationalized that tumor cells may regulate their expression differentially to their own advantage. To test if such is the case, we asked if levels of expression of Daple-fl and Daple-V2 change in response to Aspirin, a potent chemopreventive agent that reduces the risk of colorectal cancers (CRCs) by half^13,14^. We found that the treatment of DLD1 cells with low-dose Aspirin reduces both mRNA and protein for Daple-fl (Fig. 4O,P), which has both tumor-suppressive and pro-metastatic properties. By contrast, Aspirin increased the mRNA and protein for Daple-V2 (Fig. 4O,P), which has only tumor-suppressive properties. Overall, the ratio of Daple-V2 to Daple-fl was increased. When we analyzed a panel of CRC cell lines representing diverse subtypes of CRCs and harboring different mutant driver oncogenes (Supplementary Fig. 6A), we found that the Aspirin-induced changes we observed in DLD1 cells was also observed in some other CRC cell lines, but not in all of them **(**Fig. 4Q**)**. The ratio of Daple-V2:fl was increased exclusively in those cell lines which were previously shown to be most sensitive to the growth suppressive action of Aspirin^15^, and share a common driver oncogene, PIK3CA (Fig. 4Q, Supplementary Fig. 6). Whether these observed changes in Daple isoforms are due to Aspirin’s ability to inhibit cyclo-oxygenase-II (COX2), i.e., COX2-dependent or independent mechanisms^13,16^ remain unknown. Regardless, what is clear is that these changes are consistent with Aspirin’s ability to suppress polyp-to-cancer progression in the colon^17,18^, as well as its ability to inhibit metastatic progression of advanced CRCs^19–21^.

Taken together, these findings demonstrate that compared to Daple-fl, Daple-V2 may serve as a more effective inhibitor of the β-Catenin-dependent canonical Wnt pathway and tumor growth in colonies, but it does not enhance EMT or invasion **(**Fig. 4R**)**, and that their expression may be differentially regulated by tumor cells to gain advantages in growth and invasiveness. Findings also reveal that Aspirin-mediated growth suppression is associated with an increase in the Daple-V2:Daple-fl ratio, and that such a change occurs exclusively in CRC cells harboring a constitutively activated PI3K-Akt pathway.

### The anti-proliferative roles of Daple-fl and Daple-V2 are additive; simultaneous suppression of both transcripts in colon cancers carries poor prognosis

**Figure 5:**
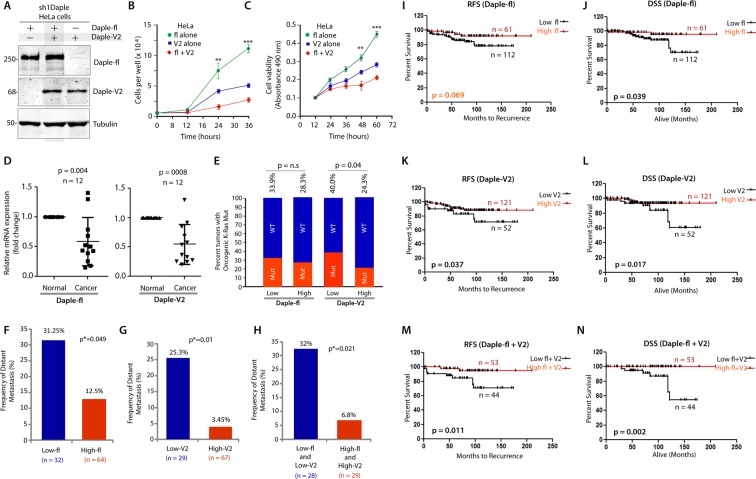
The full length (Daple-fl) and short (Daple-V2) isoforms of Daple cooperatively suppress cell proliferation and their low expression in stage II colorectal cancers carries a worse prognosis. (**A–C)** HeLa cells depleted of Daple (sh1Daple) stably expressing either Daple-fl alone, or Daple-V2 alone or both were analyzed for Daple expression by immunoblotting **(A)** and rate of cell proliferation assays **(B**,**C)**. Graphs display the rates of proliferation of various HeLa-Daple cell lines, as determined by cell counting (**B**) and cell viability assays (**C**). Results are presented as mean ± S.E.M; n = 3. **p<0.01; ***p < 0.001. (**D**) Paired colorectal tumors and their adjacent normal tissue were analyzed for relative expression of Daple isoforms by qPCR. Scatter plot displays the relative abundance of Daple expression . Error bars represent mean ± S.D. (**E**) 173 stage II colorectal cancers with known K-Ras mutant status were analyzed for levels of expression of Daple-fl and Daple-V2 mRNA by Taqman qPCR and normalized to GAPDH. Optimal cut-off values for Daple mRNA expression were statistically derived (see detailed “*Materials and Methods”*) to generate subgroups of patients with high or low expression levels. The number of tumors with or without mutant K-Ras that had either low or high expression of Daple isoforms is tabulated in Table 1. Bar graphs display the incidence (expressed as %) of K-Ras mutation (Y axis) when either Daple isoforms are either high or low. Red and blue colors indicate whether the tumors harbored oncogenic mutant or WT copy of K-Ras, respectively. The incidence of mutation is displayed on the top of each bar. Tumors with low Daple-V2 had a significant chance that they also harbor mutant K-Ras. No such relationship was seen between levels of expression of Daple-fl and mutant K-Ras. (**F–H**) Bar graphs display the incidence of distant metastasis (as %; Y axis) in stage II colorectal cancers with either low or high levels of expression of Daple-fl alone (**F**), or Daple-V2 alone (**G**), or both Daple isoforms (**H**). (**I–N**) Kaplan-Meier plot of recurrence-free (RFS) and disease-specific (DSS) survival curves of patients with stage II colorectal cancer are stratified by their levels of expression of Daple-fl alone (**I-J**), or Daple-V2 alone (**K,L**), or both Daple isoforms (**M,N**). In the RFS curves, cancers with low Daple-V2 alone exhibited decreased recurrence-free survival (**K**; significant by Log-Rank test). Although a similar trend was seen also in the case of Daple-fl (**I**), significance was not reached. Cancers with low levels of both isoforms exhibited decreased recurrence (**M**) with higher significance than each isoform alone. In the DSS curves, cancers with low Daple-fl alone or Daple-V2 alone exhibited decreased disease specific survival (**J**,**L**; significant by Log-Rank test). Cancers with low levels of both isoforms exhibited decreased survival (**N**) with higher significance than each isoform alone.

Because both Daple-fl and Daple-V2 suppress colony growth, we asked if such effects are additive. To investigate this, we carried out growth curve assessment and cell viability assays on HeLa cell lines that have been depleted of endogenous Daple by shRNA and stably expressing myc-Daple-fl or myc-Daple-V2 either alone, or together [Fig. 5A; Daple-depleted HeLa lines have been extensively characterized using a variety of approaches^1^]. In both assays we found that co-expression of Daple-fl and Daple-V2 isoforms suppressed cell growth and proliferation more effectively than compared to either isoform alone (Fig. 5B,C).

We previously showed that Daple-fl is downregulated earlier during cancer initiation (at the stage of polyp to cancer conversion), and that its expression at high levels carries a good prognosis in CRCs^1^.We also showed that levels of Daple-fl is increased later during metastatic progression and in circulating tumor cells (CTCs), and that high levels of expression carry a worse prognosis. We asked if and how the expression of Daple-V2 changes during cancer progression in the colon and what, if any, may the prognostic impact of such changes. Once again, we found several similarities and one dissimilarity. Analysis by qPCR in 12 paired colorectal tumors and their adjacent normal tissue showed that, much like Daple-fl, the expression of Daple-V2 is decreased in CRCs **(**Fig. 5D**)**. When we analyzed a cohort of patients with Duke’s Stage II CRCs, we found that tumors that express low Daple-V2 had a significantly higher incidence of oncogenic K-ras mutation **(**Fig. 5E; Table 1**)**; no such correlation was seen in the case of Daple-fl. Tumors that express low Daple-fl (Fig. 5F) or low Daple-V2 (Fig. 5G) or low levels of both isoforms **(**Fig. 5H**)** have a higher frequency of progression to distant metastases. Kaplan-Meier analyses revealed that Daple-V2 is a better prognosticator than Daple-fl (compare Fig. 5I–L) when used standalone to stratify risk for recurrence-free survival (RFS) and disease-specific survival (DSS). When accounting for high vs low levels of both Daple isoforms, an additive prognostic impact was seen compared to each alone (Fig. 5M,N). A correlation analysis showed that Daple-V2, but not Daple-fl negatively correlates with the marker for proliferative index of tumor cells Ki67^22,23^, and positive correlation with the tumor suppressor SAM and SH3-Domain Containing 1 (SASH1)^24,25^ (Table 2). Together with our findings on cell lines (growth curve, Wnt reporter and tumor cell colony formation assays), these analyses on patient tumors suggest that while both isoforms suppress tumor cell proliferation, Daple-V2 may be a more potent suppressor of tumor cell growth and proliferation than Daple-fl. These findings also define the profile of dysregulated Daple-fl and Daple-V2 expression during oncogenic progression in the colon: both isoforms are suppressed during colorectal cancer progression, and low expression levels of both isoforms alone or simultaneously exhibit decreased survival.

**Table 1:** Source data for contingency analysis (Fisher’s exact test) comparing Daple-fl or Daple-V2 expression and the presence of wild-type (WT) or oncogenic K-Ras mutation in 173 stage II colorectal carcinomas in Fig. 5E.

	Low Daple-fl	High Daple-fl
Oncogenic K-Ras Mutation	37	15
Wild-Type K-Ras	72	38
	**Low Daple-V2**	**High Daple-V2**
Oncogenic K-Ras Mutation	22	30
Wild-Type K-Ras	33	93

V2 expression and the presence of wild-type (WT) or oncogenic K-Ras mutation in 173 stage II colorectal carcinomas in Fig. 5E.

**Table 2:** Pearson’s correlation comparing Daple-fl or Daple-V2 expression and several tumor markers or histopathological parameters in 173 stage II colorectal carcinomas.

	Ki67 Index	Osteopontin	SASH1	MACC1	Age	Tumor length (cm)	Tumor differentiation	Grading
**Daple-V2**								
*r*-value	−0.1794	−0.01889	0.2041	−0.1462	0.05122	0.01046	−0.04673	0.02109
*P* value (two-tailed)	**0.0245**	0.8063	**0.0074**	0.0564	0.5059	0.892	0.5439	0.7842
**Daple-fl**								
*r*-value	0.04645	−0.03408	0.1224	−0.1131	0.02617	0.007986	−0.06434	−0.00821
*P* value (two-tailed)	0.561	0.6553	0.1077	0.1373	0.7325	0.917	0.4004	0.9147

Significant correlations are highlighted in red font. Daple-V2 showed significant negative correlation with Ki76 mitotic index and significant positive correlations with tumor suppressor SASH1 and serum levels of carcinoembryonic antigen (CEA).

Finally, we previously discovered that high levels of expression of Daple in CTCs of patients with metastatic (Duke’s Stage IV) CRCs is associated with poorer prognosis compared to those with low Daple in CTCs^7^; high Daple is associated with higher tumor recurrence at distant sites and poorer survival. Here we asked how each isoform contributed to the prognostic impact using the same cohort. We found that Daple-fl,but not Daple-V2, expression is increased in disseminated tumor cells compare to healthy subjects **(**Supplemental Fig. 7A,B**)**. When we assessed their prognostic impact on survival, we found that although high expression of each isoform correlated with worse PFS **(**Supplementary Fig. 7C,F**)**, only Daple-fl levels carried a prognostic impact for DSS and overall survival (OS) **(**Supplementary Fig. 7D,E,G,H**)**. These findings are in keeping with our prior observations that pro-invasive and pro-EMT signatures are triggered exclusively by Daple-fl, but not Daple-V2 (Fig. 2Q).

From these results we deduce that both Daple-fl and Daple-V2 cooperatively suppress tumor cell proliferation, that both transcripts are reduced during adenoma-to-carcinoma progression, and that the two isoforms have an additive prognostic impact, in that their reduced expression in tumors carries a poor prognosis. However, the two isoforms differ in their ability to trigger EMT and invasion; Daple-fl, but not Daple-V2 can support that.

### Daple is downregulated during adenoma-to-carcinoma conversion

**Figure 6:**
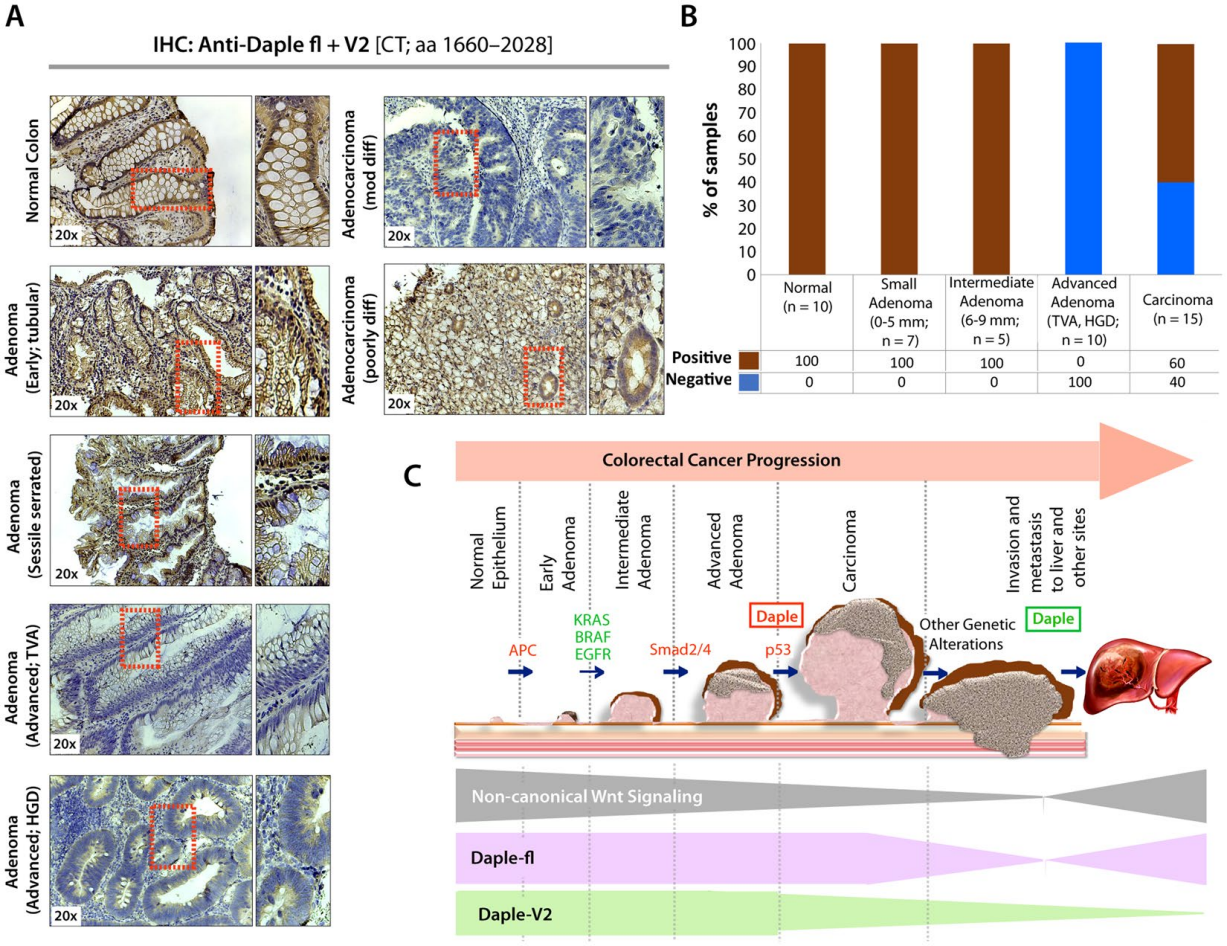
Both Daple-fl and Daple-V2 isoforms are downregulated during adenoma-to-carcinoma conversion. (**A,B**) Expression of Daple protein was analyzed in formalin-fixed paraffin embedded human tissues (normal, adenomas and carcinomas) by immunohistochemistry (IHC) using anti-Daple-CT antibody that can detect both Daple-fl and Daple-V2 isoforms. *Left*: Representative tissues from each stained category are shown. Brown = positive stain. *Right*: Bar graphs display the proportion of samples in each category that stained positive vs. negative. **(C)** Schematic summarizing profile of expression of Daple-fl and Daple-V2 isoforms during cancer initiation and metastatic progression in the colon. *Upper*: Various steps and histopathological stages of colorectal cancer progression are shown. Major genetic mutations/ deletions of key genes that herald the step-wise progression are indicated. Daple (both Daple-fl and Daple-V2) are decreased during adenoma to carcinoma progression (red box). Later, during cancer progression and systemic dissemination, total levels of Daple go up (green box), largely owing to an upregulation of its full-length (Daple-fl) transcript. *Lower*: Changes in the profile of expression of both Daple isoforms (Daple V2 = green; Daple-fl = purple) and their relationship to the previously identified patterns of non-canonical Wnt signaling (gray) is shown.

Next we asked if suppression of Daple-fl and Daple-V2 transcripts during adenoma-to-cancer progression is also associated with reduced Daple proteins. Using an antibody raised against the C-terminus of Daple, which is identical between Daple-fl and Daple-V2, we confirmed that total Daple is expressed in the normal colon and in early and intermediate adenomas, but it is suppressed in advanced adenomas with villous features or high-grade dysplasia **(**Fig. 6A,B**)**. In cancers, ~60% expressed Daple, but ~40% did not.

Taken together, our findings support the following model (Fig. 6C). While both Daple-fl and Daple-V2 isoforms serve as growth suppressors early during oncogenesis, only Daple-fl serves as a pro-metastatic protein later during cancer progression. While both are suppressed during the step of polyp-to-cancer conversion, only Daple-fl is induced later during cancer invasion and dissemination.

## Conclusions

The major discovery we report here is the identification and characterization of a novel physiologic isoform of Daple, Daple-V2, which appears to contain the minimal modules that enable Daple to antagonize canonical β-Catenin-dependent Wnt signals and inhibit tumor cell growth and proliferation. Both isoforms are reduced during polyp-to-cancer progression in the colon. Compared to Daple-fl, Daple-V2 appears to be a more potent tumor suppressor and a better prognostic marker in primary tumors.

What Daple-V2 lacks is the ability to trigger EMT and invasion, which is a feature unique to Daple-fl. Consistent with these findings, we also found that although both isoforms collaboratively suppress tumor cell growth, and have an additive prognostic impact, only Daple-fl is increased in invasive margins and in CTCs disseminated during metastatic progression. Because we previously showed that a functional GBA motif is essential for Daple-fl to trigger EMT and invasion^1^, that Daple-V2 cannot trigger EMT despite a functionally intact GBA suggests that these functions not only require the coupling of Daple’s C-terminus to G-proteins, but also the functions of its N-terminal HOOK and coiled-coil domains, e.g., phosphoinositide (PI3P) binding and localization to the pericentriolar recycling endosomes^11^, or binding to the dynein-dynactin motor complex^26^.

Although it remains unknown how cells modulate the expression of each Daple isoform, our studies provided precious clues. When exposed to the chemoprevention drug, Aspirin, CRC cells that are sensitive to its growth-suppressive action were able to suppress Daple-fl but concomitantly increased Daple-V2; this effect was observed only in CRC cell lines that harbor the PIK3CA mutation. Prior clinical trials in humans^27,28^ and studies on cultured cells^15,29^ have confirmed that CRCs/cells that harbor PIK3CA mutation are most sensitive to the growth-suppressive action of Aspirin. Based on the properties of each isoform, we conclude that such a tradeoff is expected to overall maximize the desirable tumor-suppressive effect of Daple without the undesirable pro-EMT and pro-invasive effects. In fact, a decrease in the levels of Daple-V2 with a concomitant increase in Daple-fl transcripts in the circulating blood has recently been shown to correlate with increased stemness markers, an aggressive disease course and a shorter relapse-free survival in patients with melanoma^30^.

Despite the insights gained, a lot remains unknown. For example, it is possible that the mRNA for Daple-V2 isoform could have cellular functions independently of protein expression. Similarly, the numerous other splice variants, including those not interrogated here, may contribute to a rich complex of Daple biology in cancer.

In conclusion, we have revealed one of perhaps many ways how expression of Daple and its functions are regulated in the colon. Our findings that Daple isoforms can be pharmacologically manipulated to selectively augment its tumor-suppressive functions, while suppressing its pro-metastatic functions raises hope that therapeutic strategies could be tailored to meet such goals.

## Materials and Methods

### Reagents and antibodies

Unless otherwise indicated, all reagents were of analytical grade and obtained from Sigma-Aldrich. Cell culture media were purchased from Invitrogen. All restriction endonucleases and Escherichia coli strain DH5α were purchased from New England Biolabs. E. coli strain BL21 (DE3) and phalloidin-Texas Red were purchased from Invitrogen. Genejuice transfection reagent was from Novagen. PfuUltra DNA polymerase was purchased from Stratagene. Recombinant Goat anti-rabbit and goat anti-mouse Alexa Fluor 680 or IRDye 800 F(ab′)2 used for immunoblotting were from Li-Cor Biosciences. Mouse anti-α-tubulin and anti-actin were obtained from Sigma; anti-Myc was obtained from Covance, and anti-GFP from Santa Cruz Biotechnology. Rabbit anti–pan-Gβ (M-14), and anti-Gαi3 were obtained from Santa Cruz Biotechnology. Anti-Daple antibodies were generated in collaboration with Millipore using the C-terminus of Daple (1660–2028 aa) as an immunogen. WNT proteins were purified from 6 liters of CHO CM. CM was complemented with 1% Triton X-100 (vol/vol), 20 mM Tris-Cl pH 7.5, and 0.01% NaN_3_.

### Plasmid constructs and mutagenesis

Cloning of N-terminally tagged myc-Daple-fl and mCherry Daple-fl was carried out as described previously^1,11^. The subsequent site-directed mutagenesis and truncated constructs (myc-Daple full length F1675A (FA) and deleted from 2025–2028 aa (ΔPBM) were carried out on this template using Quick Change as per manufacturer’s protocol. Cloning of N-terminally tagged myc-Daple-V2 was carried out by PCR cloning directly from HeLa cDNA using primers containing the unique 5′ region (MSVLS) in Daple V2 sequence (corresponding to UniProtKB - Q9P219-2) and being inserted into myc-pcDNA 3.1 (+) between Kpn-1/EcoR1. To clone mCherry Daple-V2, Daple-V2 was PCR amplified from HeLa cDNA with primers flanked with recombination site for Gateway cloning system (Invitrogen), as previously described^11^. Daple-V2-FA (F194A) and Daple-V2- ΔPBM (deleted from 549–552aa) were also directly cloned out from pcDNA3.1(+)-Daple-fl-FA and pcDNA3.1(+)-daple-fl- ΔPBM. Cloning of rat Gα-proteins into pGEX-4T-1 GST-Gαi3 has been described previously^31–35^. GST-tagged FZD7-CT construct^36^ was a generous gift from Ryoji Yao (JFCR research institute, Japan). GST-Dvl2-PDZ was from Raymond Habas (Temple University, Philadelphia, PA).

### Protein expression and purification

GST, GST-Gαi3, GST-PDZ and GST-FZD7 fusion constructs were expressed in *E*. *coli* strain BL21 (DE3) (Invitrogen) and purified as described previously^31–33^. Briefly, bacterial cultures were induced overnight at 25 °C with 1 mM isopropyl β-D-1-thiogalactopyranoside (IPTG). Pelleted bacteria from 1 L of culture were re-suspended in 10 ml GST-lysis buffer [25 mMTris-HCl, pH 7.5, 20 mMNaCl, 1 mM EDTA, 20% (v:v) glycerol, 1% (v:v) Triton TX-100, 2X protease inhibitor cocktail (Complete EDTA-free, Roche Diagnostics]. After sonication (4 × 20s, 1 min between cycles), lysates were centrifuged at 12,000 g at 4 °C for 20 min. Except for GST-FZD (see *in vitro* GST pulldown assay section), solubilized proteins were affinity purified on glutathione-Sepharose 4B beads (GE Healthcare). Proteins were eluted, dialyzed overnight against PBS and stored at −80 °C.

### Cell culture and the rationale for choice of cells in various assays

Tissue culture was carried out essentially as described before^31,32,37^. We used a total of 3 different cell lines in this work, each chosen carefully based on its level of endogenous Daple expression and the type of assay. All these cell lines were cultured according to ATCC guidelines. Cos7 cells were primarily used for transient overexpression of tagged Daple protein and lysates of these cells were used as source of proteins in pulldown assays. We chose to carry out these assays in Cos7 cells because they are easily and efficiently transfected (>90% efficiency) with most constructs. The added advantage is that they have no detectable endogenous Daple and provide a system to selectively analyze the properties of WT vs mutant Daple constructs without interference from endogenous Daple.

DLD1 were primarily used to study the effect of Daple on cancer cell growth properties (anchorage-dependent) and to assess the effect of Daple on the classical Wnt signaling pathway (βCatenin/TCF/LEF). There are several reasons why this cell line was chosen: (1) We focused on colorectal cancer in this study and DLD1 cells were appropriate to translate our findings because they are human colorectal cancer cells; (2) We determined that levels of Daple are significantly lower/undetectable (~10 fold) in these cells compared to normal colon (data not shown), thereby allowing us to reconstitute Daple expression exogenously and analyze the effect of various mutant Daple constructs without significant interference due to the endogenous protein; (3) These cells have been extensively characterized with respect to most oncogenes (ATCC database), and are highly tumorigenic in 2-D and 3-D cultures due to a mutation in KRAS (G13D) (Shirasawa *et al*., 1993; Ahmedet al., 2013); (4) They are a sensitive model to study how various manipulations of the noncanonical Wnt signaling pathway oppose the canonical Wnt pathway during tumor growth because they constitutively secrete Wnt ligands to maintain high levels of the canonical signaling^38^ within the growth matrix. Production and secretion of endogenous ligands bypasses the need to add exogenous ligands repeatedly during prolonged assays that last ~2 weeks.

Low passage NIH3T3 fibroblasts were used exclusively in 3-D Matrigel invasion. The rationale for their use in invasion assay lies in the fact that non-transformed NIH3T3 fibroblasts are poorly invasive in vitro and non-tumorigenic and non-metastatic in animal studies^39–42^. It is because of this reason, NIH3T3 cells are widely used to study proteins that can trigger a gain in invasive properties^43^. The rationale for using NIH3T3 in the above assays is further strengthened by the fact that they are highly transfectable (~80% transfection efficiency with myc-Daple) and express Daple at very low endogenous levels (as determined by immunoblotting and qPCR) compared to normal colonic epithelium. Such expression pattern allows us to study the effect of various Daple mutant constructs without significant interference due to the endogenous protein. Multiple other CRC cell lines used in this work were obtained from ATCC and cultured as recommended.

### Transfection; generation of stable cell lines and cell lysis

Transfection was carried out using Genejuice (Novagen) for DNA plasmids following the manufacturers’ protocols. DLD1 cell lines stably expressing Daple constructs were selected after transfection in the presence of 800 μg/ml G418 for 6 weeks. The resultant multiclonal pool was subsequently maintained in the presence of 500 μg/ ml G418. Daple expression was verified independently using anti-Daple antibody by immunoblotting and estimated to be ~5x the endogenous level.

### Quantitative immunoblotting

For immunoblotting, protein samples were separated by SDS-PAGE and transferred to PVDF membranes (Millipore). Membranes were blocked with PBS supplemented with 5% non-fat milk (or with 5% BSA when probing for phosphorylated proteins) before incubation with primary antibodies. Infrared imaging with two-color detection and band densitometry quantifications were performed using a Li-Cor Odyssey imaging system exactly as done previously^32,34,35,37,44^. Full length blots are provided in Fig. 2 and Supplementary Fig. 4 for endogenous and overexpressed exogenous Daple constructs. For all other instances, PVDF membrane was cut into strips for incubation with various antibodies (primary and secondary) to rigorously examine all antigens (phospho vs total target protein and loading controls) on the same gel/membrane to be reflective of balanced loading of samples. Cropped gels are indicated by borders. All Odyssey images were processed using Image J software (NIH) and assembled into figure panels using Photoshop and Illustrator software (Adobe).

### Isoform expression analysis

These analyses were carried out using a web server, ISOexpresso^45^, which facilitates expression-based isoform-level analysis of RNA sequencing data from 9,690 tumors and 732 normal controls of 30 cancer types from TCGA (The Cancer Genome Atlas) Data Portal. Transcripts are matched to gene and isoform information based on hg19/GRCh37 including IDs of genes and isoforms, genomic location, and known canonical/principal isoforms from UCSC Annotation database, UniProt (Universal Protein Resource), RefSeq (NCBI Reference Sequence Database),Ensembl, CCDS (Consensus CDS), APPRIS (Annotating principal splice isoforms), and HGNC (HUGO Gene Nomenclature Committee).We performed the analysis using the server’s ‘*Isoform Expression View* function’ which allows the analysis of tissue-specific RNA expression patterns of isoforms by selecting one or more tissues (cancer types) and querying a gene of interest.

### *In vitro* GST pulldown

Purified GST alone, GST-Gαi3 or GST-PDZ (5 µg) were immobilized on glutathione-Sepharose beads and incubated with binding buffer [50 mM Tris-HCl (pH 7.4), 100 mM NaCl, 0.4% (v:v) Nonidet P-40, 10 mM MgCl_2_, 5 mM EDTA, 30 µM GDP, 2 mM DTT, protease inhibitor mixture] for 90 min at room temperature as described before^31,32,37,46^. Lysates (~250 µg) of Cos7 cells expressing appropriate myc-Daple-V2 constructs were added to each tube, and binding reactions were carried out for 4 h at 4 °C with constant tumbling in binding buffer [50 mM Tris-HCl (pH 7.4), 100 mM NaCl, 0.4% (v:v) Nonidet P-40, 10 mM MgCl_2_, 5 mM EDTA, 30 µM GDP, 2 mM DTT]. Beads were washed (4x) with 1 mL of wash buffer [4.3 mM Na_2_HPO_4_, 1.4 mM KH_2_PO_4_ (pH 7.4), 137 mM NaCl, 2.7 mMKCl, 0.1% (v:v) Tween 20, 10 mM MgCl_2_, 5 mM EDTA, 30 µM GDP, 2 mM DTT] and boiled in Laemmli’s sample buffer. Immunoblot quantification was performed by infrared imaging following the manufacturer’s protocols using an Odyssey imaging system (Li-Cor Biosciences).

GST-FZD7-CT construct was immobilized on glutathione-Sepharose beads directly from bacterial lysates by overnight incubation at 4 °C with constant tumbling as described before^1^. Next morning, GST-FZD7-CT immobilized on glutathione beads were washed and subsequently incubated with His-tagged Daple-CT or Gαi3 proteins at 4 °C with constant tumbling. Washes and immunoblotting were performed as previously.

### *β-Catenin* reporter assays

These assays were carried out using the well-established reporter 7xTcf-eGFP(7TGP)^47^. Stable cells lines expressing this reporter were generated by lentiviral transduction and subsequent selection using standard procedures. Lentiviral infection and selection were performed according to standard procedures. Briefly, 10 cm plates DLD1 cells at 70% confluency were incubated with media containing 8 µg/mL polybrene and 10 µl of lentivirus for 6 h. After 24 hours post infection, selection of puromycin-resistant clones was initiated by adding the antibiotic at 2 µg/ml final concentration. The resultant DLD1-7TGP stable cells were subsequently transfected with various myc-Daple V2 constructs and selected for G418 resistance as described earlier in methods. The DLD1-7TGP cells stably expressing myc-Daple were incubated overnight at 0.2% FBS, analyzed by fluorescence microscopy, and photographed prior to lysis. Whole cell lysates samples were then boiled in Laemmli’s sample buffer and GFP protein expression was monitored by immunoblotting.

### Cell proliferation assays

Two complementary methods were used. For cell counting assays, cells were seeded at 1 × 104 per well in Ham’s F12 media with 10% (v/v) FBS and grown at 37 °C in a humidified 5% CO_2_ incubator. At various times cells were trypsin released and manually counted with a haemocytometer. Results are means ± SEM (n = 3). For cell viability assays, cells were seeded into 96-well plates at 2 × 10^5^ cells/ml and cultured in DMEM/Ham’s F12 media supplemented with 2% FBS for 1–5 days. Cell growth was documented every 24 hours via a colorimetric assay using a 3-(4,5-dimethylthiazol-2-yl)-2,5-diphenyltetrazolium bromide (MTT) assay (Sigma). Absorbance values were collected at 490 nm using a SpectraMax 190 microplate reader (Molecular Devices, Sunnyvale, CA, USA). Control samples were treated with vehicle (0.1% DMSO or ethanol in DMEM/Ham’s F12 culture media). In each individual experiment, proliferation was determined in triplicate, and the overall experiment was repeated at least three times.

### Anchorage-dependent colony growth assay

Anchorage-dependent growth was monitored on solid (plastic) surface. Approximately ~1000 DLD1 cells stably expressing various Daple constructs were plated in 6-well plates and incubated in 5% CO_2_ at 37 °C for ~2 weeks in 0.2% FBS growth media. Colonies were then stained with 0.005% crystal violet for 1 h. The remaining DLD1 cells were lysed and analyzed by WB to confirm Daple construct expression. Each experiment was analyzed in triplicate.

### Invasion assays

NIH3T3 cell invasion assay in 3D culture was performed according to the manufacturer’s protocol (Trevigen, Cultrex 3D Spheroid BME Cell Invasion Assay, catalog #3500–096-K). Briefly, NIH3T3 cells (3000 cells) transfected with empty vector (control) or myc-Daple constructs were incubated first in the Spheroid Formation extracellular matrix (ECM) containing 0.2% FBS for 3 days. Invasion matrix was then added and layered on top with media containing FBS. Serum-triggered cell invasion was photographed under light microscope every day for 10 days and fresh media (FBS concentration is increased each time in order to maintain a gradient) was replenished every 48 hours. Photographs were analyzed and pseudocolored by Image J to reflect cell density.

### Patient cohort for mRNA analysis

The ethics committee of the Klinikumrechts der Isar, Munich, Germany, approved collection of the patient samples (#1926/07, and #5428/12). All samples were obtained after prior informed written consent. Tumor tissue from 173 patients with histopathologically confirmed stage II (AJCC/UICC) colon cancer who underwent complete surgical resection (R0) between 1987 and 2006 was obtained, by a pathologist immediately after surgical resection. Specimens were transferred into liquid nitrogen and stored at −80 °C until further processing. None of the patients received neoadjuvant treatment. No metachronous tumors were found in the colon or rectum. As reported previously in detail, clinical data and post-operative follow-up was collected for all patients; moreover, DNA was isolated for KRAS and BRAF mutation analysis, as well as microsatellite instability testing^48^. Total mRNA was extracted by standard procedures (Qiagen, Hilden, Germany), after histology guided sample selection to ensure a tumor cell content of >50%, and transcribed to cDNA as described in detail earlier, for expression analysis of DAPLE^48^.These studies abide by the Declaration of Helsinki principles.

### RNA isolation, standard curve and quantitative PCR (qPCR)

Total RNA was isolated using an RNeasy kit (QIAGEN) as per the manufacturers’ protocol. First-strand cDNA was synthesized using Superscript II reverse transcriptase (Invitrogen), followed by ribonuclease H treatment (Invitrogen) prior to performing quantitative real-time PCR. A standard curve, to quantify mRNA copy number, was constructed using larger PCR products (~700 bp) that included the target sequence used in qPCR. Reactions omitting reverse transcriptase were performed in each experiment as negative controls. Reactions were then run on a real-time PCR system (ABI StepOnePlus; Applied Biosystems). Gene expression was detected with SYBR green/Taqman assay (Invitrogen), and relative gene expression was determined by normalizing to GAPDH using the comparative ΔCt/Relative standard curve method.

Primer and probe sequences are as listed below.

**Probes and primers used in verifying Daple isoforms (**Fig. 1B**)**

Primer 1: ACCTGCCAAGAAAGAAGG

Primer 2: ACCTGCAGTGGCAATGGC

**Probes and primers used in Taqman assays:** for human tumor/tissue and CTC samples

GAPDH:

hGAPDH-fwd: 5′-CAGTTGTAGGCAAGCTGCGA-3′

hGAPDH-rev: 5′-TATGACAGGCCCGAAGCTTCT-3′

hGAPDH-probe: 5′-CCAAGCCTGAGGGCAAGGCTATAATAGATGAAT-3′

hGapdh-standard fwd: 5′-GCT GTG ACA TCA GGG CAA T-3′

hGapdh-standard rev: 5′-GGC GGT GGT GGC TTT ATT T-3′

Daple-fl:

hDaple-fl-fwd: 5′-CGGGACCTCACCAAGCAA-3′

hDaple-fl-rev: 5′-CTGCTGAGCTGCTGGCTCTT-3′

hDaple-fl-probe: 5′-CAACTCTGAGGGAGGACCTGGTGCTC-3′

hDaple-fl-Standard-fwd: 5′-GGATGCAGTCTTGGACGATAG-3′

hDaple-fl-Standard-rev: 5′-CTTCTTTCATGGCTAGTGTTGTTT-3′

Daple-V2:

hDaple-V2-fwd: 5′-GGAGCCTCAGGATATACGTGCA-3′

hDaple-V2-rev: 5′-TCAAGGCTGCCTCTGTGTGG-3′

hDaple-V2-probe: 5′-CAGGATGTCCGTACTAAGCCCTGGGGATC-3′

hDaple-V2 Standard-fwd: 5′-CACTCCCTGGACCATTTCTT-3′

hDaple-V2 Standard-rev: 5′-CTGTAGTGGTGGCTGAAGTT-3′

**Primers used in SYBR-green assays:** for cell-based analyses

Lox-3:

hq-LOXL3 fwd: 5′-ATGGGTGCTATCCACCTGAG-3′

hq-LOXL3 rev: 5′-GAGTCGGATCCTGGTCTCTG-3′

Axin-2:

hAxin-2-fwd: 5′-GAGTGGACTTGTGCCGACTTCA-3′

hAxin-2-rev: 5′-GGTGGCTGGTGCAAAGACATAG-3′

Vimentin:

hVim-fwd: 5′-AAGAGAACTTTGCCGTTGAA-3′

hVim-rev: 5′-GTGATGCTGAGAAGTTTCGT-3′

SFRP-1:

hSFRP-1-fwd: 5′-GAGTTTGCACTGAGGATGAAAA-3′

hSFRP-1-rev: 5′-GCTTCTTCTTCTTGGGGACA-3′

GADPH:

hGADPH q-fwd2: 5′-TCA GTT GTA GGC AAG CTG CGA CGT-3′

hGADPH q-rev2: 5′-AAGCCAGAGGCTGGTACCTAGAAC-3

DAPLE:

hDaple-fl-fwd: 5′-TGA CAT GGA GAC CCT GAA GGC TGA-3′

hDaple fl-rev2: 5′-TTTCATGCGGGCCTCACTGCTGA-3′

hDaple V2-Fwd: 5′-GTT GTC ACA CTC CCT GGA CCA TTT C-3′

hDaple V2-Rev: 5′-GCTTTGGTTTTAGATCCCCAGGGC-3′

### Patient cohort for IHC analysis

Formalin-fixed paraffin embedded (FFPE) normal, polyp and cancer tissues used in this study were obtained from patients undergoing routine colonoscopies and provided by the section of Gastroenterology, VA San Diego Healthcare System. The protocol was approved by the Human Research Protection Program Institutional Review Board (IRB; protocol H130266). These studies abide by the Declaration of Helsinki principles.

### Immunohistochemistry

Colon specimens of known histologic type were analyzed by IHC using a previously-validated anti-Daple-CT rabbit polyclonal antibody raised against Daple (1660–2028 aa) (1:50; Millipore Inc.). Briefly, formalin-fixed, paraffin-embedded tissue sections of 4 µm thickness were cut and placed on glass slides coated with 3-aminopropyl triethoxysilane, followed by deparaffinization and hydration. Heat-induced epitope retrieval was performed using citrate buffer (pH 6) in a pressure cooker. Tissue sections were incubated with 3% hydrogen peroxidase for 15 min to block endogenous peroxidase activity, followed by incubation with primary antibodies overnight in a humidified chamber at 4 °C. Immunostaining was visualized with a labeled streptavidin-biotin using 3,3′-diaminobenzidine as a chromogen and counterstained with hematoxylin. All the samples were first quantitatively analyzed and scored based on 2 independent criteria. First, the intensity of staining was scored on a scale of 0 to 3, where 0 = no staining, 1 = light brown, 2 = brown, and 3 = dark brown. Second, the percentage of the cells that stained positive in the tumor area was scored on a scale of 0 to 4, where 0 = 0, 1 = ≤10%, 2 = 11–50%, 3 = 51–75%, and 4 = >75%. Subsequently, each tumor sample was assigned a final score, which is the product of its (intensity of staining) × (% cells that stained positive). Tumors were categorized as negative when their final score was <3 and as positive when their final score was ≥3.

### Statistical analyses

Statistical evaluation was performed using GraphPad Prism 5 software. Unless stated otherwise, statistical significance was determined using Student’s *t* test. The associations between the expression level of Daple isoforms and K-ras mutation status and metastatic status of disease were investigated by Fisher’s exact test. Parametric Pearson’s correlation and nonparametric Spearman’s correlation analysis were used to assess the relationship between the expression of Daple isoforms and patients’ age, tumor size, differentiation and grading, the level of Ki67-Index, Osteopontin, SASH1, MACC1 and CEA. In order to derive optimal cut-off values of gene expression levels, maximally selected log-rank statistics performed by Cutoff Finder (R Software version 2.15.0.)^49^ were used. Time-dependent survival probabilities were estimated with the Kaplan-Meier method using the log-rank test. All statistical tests were performed two-sided, and p- values less than 0.05 were considered to be statistically significant.

## Supplementary information

Supplementary Figures and Legends
