# Supplementary material for: Two Isoforms of the Guanine Nucleotide Exchange Factor, Daple/CCDC88C Cooperate as Tumor Suppressors
Source: Sci Rep. 2019 Aug 20;9:12124. doi: 10.1038/s41598-019-48420-w (PMC6702192; doi:10.1038/s41598-019-48420-w)
Supplement: Supplementary file 1 — Supplementary Figures and Legends [file 41598_2019_48420_MOESM1_ESM.pdf]

SUPPLEMENTARY ONLINE MATERIALS

Two Isoforms of the Guanine Nucleotide Exchange Factor, Daple/CCDC88C Cooperate as Tumor Suppressors

Jason Ear[1, 2¶], Ying Dunkel[1¶], Yash Mittal[1], Blaze B. C. Lim[1], Lawrence Liu[1], Magda K. Holda[1], Ulrich Nitsche[3], Jorge Barbazán[4], Ajay Goel[5], Klaus-Peter Janssen[3], Nicolas Aznar[1, 6¶§], and Pradipta Ghosh[1,2, 7§]

[1]*Department of Medicine, University of California, San Diego, La Jolla, California, USA.*

[2]*Department of Cellular and Molecular Medicine, University of California, San Diego, La Jolla, California, USA.*

[3]*Department of Surgery, Klinikumrechts der Isar, TechnischeUniversitätMünchen, Munich, Germany.*

[4]*Translational Medical Oncology Laboratory, Health Research Institute of Santiago (IDIS), SERGAS. Santiago de Compostela, Spain.*

[5]*Division of Gastroenterology, Department of Internal Medicine and Charles A. Sammons Cancer Center and Baylor Research Institute, Baylor University Medical Center, Dallas, Texas, USA.*

[6]*Department of Cancer Cell Plasticity, Team Development Cancer and Stem Cells, Cancer Research Center of Lyon, Centre Léon Bérard, Lyon, France.*

[7]*Moores Cancer Center, University of California, San Diego, La Jolla, California, USA.*

Running Title: Two Daple isoforms cooperatively suppress tumorigenesis

Key Words: G protein, Wnt, Frizzled, ccdc88c/DAPLE, βCatenin, Disheveled, PI3-Kinase, Akt, Rac1, Colon cancer.

¶ These authors contributed equally to this work.

§ **To whom correspondence should be addressed**: §Corresponding author. Email: Nicolas.AZNAR@lyon.unicancer.fr (N.A.), prghosh@ucsd.edu (P.G.).

A

Name	Protein Length	Ensembl (Transcript ID)	UniProt (Identifier)	RefSeq
Full Length	2028 aa	ENST00000389857.10 (GRCh38)	Q9P219-1	NM_001080414
V2	552 aa	ENST00000331194.7 (GRCh37)	Q9P219-2	
V3	506 aa	ENST00000331194.8 (GRCh38)	A0A0A0MR69	
V4	478 aa	–	Q9P219-3	
V5	96 aa	ENST00000553403.1 (GRCh38)	G3V3S0	
V6	88 aa	ENST00000389856.9 (GRCh38)	Q0P665	
V7	243 aa	–	B4DZB8	

B

```
Full_Length  DPASPAASQPLRSQAENPDTPALGSNCAEERDAHNGSVGKGPGDLKPKRGSPHRGSLDRT  1500
V2           ------------------------------------MSVLSPGDLKPKRGSPHRGSLDRT  24
V3           ------------------------------------MSVLSPGDLKPKRGSPHRGSLDRT  24
V4           ------------------------------------------------------------  0
                                           Unique V2 and V3 N-terminus

Full_Length  DASTDLAMRSWPSELGSRTCSTSATTTAPSNSTPIARHPGRTKGYNSDDNLCEPSLEFEV  1560
V2           DASTDLAMRSWPSELGSRTCSTSATTTAPSNSTPIARHPGRTKGYNSDDNLCEPSLEFEV  84
V3           DASTDLAMRSWPSELGSRTCSTSATTTAPSNSTPIARHPGRTKGYNSDDNLCEPSLEFEV  84
V4           --------------------------------MPSS------------------TLPGWP  10
                                              * :  Unique V4 N-terminus  :*

Full_Length  PNHRQYVSRPSSLESSRNTSSNSSPLNLKGSSEQLHGRSESFSSEDLIPSRDLATLPREA  1620
V2           PNHRQYVSRPSSLESSRNTSSNSSPLNLKGSSEQLHGRSESFSSEDLIPSRDLATLPREA  144
V3           PNHRQYVSRPSSLESSRNTSSNSSPLNLKGSSEQLHGRSESFSSEDLIPSRDLATLPREA  144
V4           GSSGGPVSRPSSLESSRNTSSNSSPLNLKGSSEQLHGRSESFSSEDLIPSRDLATLPREA  70
              .    ******************************************************

Full_Length  STPGRNALGRHEYPLPRNGPLPQEGAQKRGTAPPYVGVRPCSASPSSEMVTLEEFLEESN  1680
V2           STPGRNALGRHEYPLPRNGPLPQEGAQKRGTAPPYVGVRPCSASPSSEMVTLEEFLEESN  204
V3           STPGRNALGRHEYPLPRNGPLPQEGAQKRGTAPPYVGVRPCSASPSSEMVTLEEFLEESN  204
V4           STPGRNALGRHEYPLPRNGPLPQEGAQKRGTAPPYVGVRPCSASPSSEMVTLEEFLEESN  130
             ************************************************************
                                                                GBA Motif

Full_Length  RSSPTHDTPSCRDDILSDYFRKASDPPAIGGQPGPPAKKEGAKMPTNFVAPTVKMAAPTS  1740
V2           RSSPTHDTPSCRDDILSDYFRKASDPPAIGGQPGPPAKKEGAKMPTNFVAPTVKMAAPTS  264
V3           RSSPTHDTPSCRDDILSDYFRKASDPPAIGGQPGPPAKKEGAKMPTNFVAPTVKMAAPTS  264
V4           RSSPTHDTPSCRDDILSDYFRKASDPPAIGGQPGPPAKKEGAKMPTNFVAPTVKMAAPTS  190
             ************************************************************

Full_Length  EGRPLKPGQYVKPNFRLTEAEAPPSVAPRQAQPPQSLSLGRPRQAPVPPASHAPASRSAS  1800
V2           EGRPLKPGQYVKPNFRLTEAEAPPSVAPRQAQPPQSLSLGRPRQAPVPPASHAPASRSAS  324
V3           EGRPLKPGQYVKPNFRLTEAEAPPSVAPRQAQPPQSLSLGRPRQA---------------  309
V4           EGRPLKPGQYVKPNFRLTEAEAPPSVAPRQAQPPQSLSLGRPRQAPVPPASHAPASRSAS  250
             *********************************************

Full_Length  LSRAFSLASADLLRASGPEACKQESPQKLGAPEALGGRETGSHTLQSPAPPSSHSLARER  1860
V2           LSRAFSLASADLLRASGPEACKQESPQKLGAPEALGGRETGSHTLQSPAPPSSHSLARER  384
V3           -------------------------------PEALGGRETGSHTLQSPAPPSSHSLARER  338
V4           LSRAFSLASADLLRASGPEACKQESPQKLGAPEALGGRETGSHTLQSPAPPSSHSLARER  310
                                            *****************************

Full_Length  TPLVGKAGSSCQGPGPRSRPLDTRRFSLAPPKEERLAPLHQSATAPAIATAGAGAAAAGS  1920
V2           TPLVGKAGSSCQGPGPRSRPLDTRRFSLAPPKEERLAPLHQSATAPAIATAGAGAAAAGS  444
V3           TPLVGKAGSSCQGPGPRSRPLDTRRFSLAPPKEERLAPLHQSATAPAIATAGAGAAAAGS  398
V4           TPLVGKAGSSCQGPGPRSRPLDTRRFSLAPPKEERLAPLHQSATAPAIATAGAGAAAAGS  370
             ************************************************************

Full_Length  GSNSQLLHFSPAAAPAARTKPKAPPRSGEVATITPVRAGLSLSEGDGVPGQGCSEGLPAK  1980
V2           GSNSQLLHFSPAAAPAARTKPKAPPRSGEVATITPVRAGLSLSEGDGVPGQGCSEGLPAK  504
V3           GSNSQLLHFSPAAAPAARTKPKAPPRSGEVATITPVRAGLSLSEGDGVPGQGCSEGLPAK  458
V4           GSNSQLLHFSPAAAPAARTKPKAPPRSGEVATITPVRAGLSLSEGDGVPGQGCSEGLPAK  430
             ************************************************************

Full_Length  SPGRSPDLAPHLGRALEDCSRGSVSKSSPASPEPGGDPQTVWYEYGCV  2028
V2           SPGRSPDLAPHLGRALEDCSRGSVSKSSPASPEPGGDPQTVWYEYGCV  552
V3           SPGRSPDLAPHLGRALEDCSRGSVSKSSPASPEPGGDPQTVWYEYGCV  506
V4           SPGRSPDLAPHLGRALEDCSRGSVSKSSPASPEPGGDPQTVWYEYGCV  478
             ************************************************ PDZ-binding motif
```

Supplementary Figure 1: **(A)** Table shows the identifiers (Ensembl, UniProt, and RefSeq) of the transcripts and protein length encoded by the Daple/CCDC88C gene. Daple-V2 was annotated in Ensembl assembly GRCh37 and Daple-V3 was annotated on GRCh38. **(B)** Sequence alignment of the various isoforms containing the modular C-terminal domain and its various motif are displayed. Red Box = Unique residues at the N-terminal end of Daple-V2 and Daple-V3. Blue Box = Unique residues at the N-terminal end of Daple-V4. Brown = GBA motif identified previously[1]. Green Box = PDZ-binding motif (PBM) that was reported earlier[2].

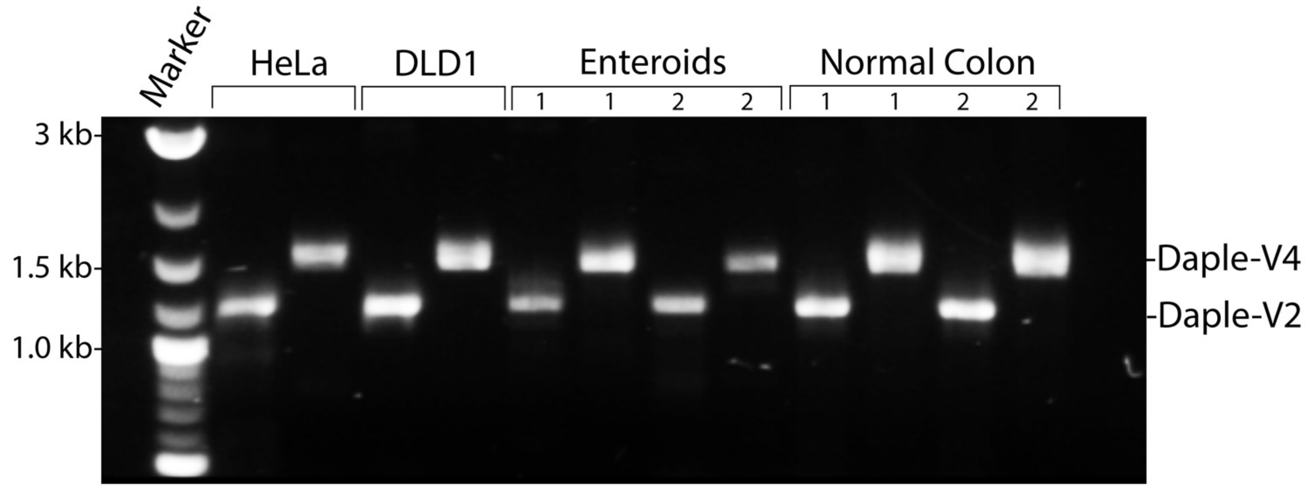

Supplementary Figure 2: Semi-quantitative RT-PCR for Daple-V4 and Daple-V2 transcripts from HeLa and DLD1 cell lines, human gut enteroids, and normal human colon. Two independent samples of gut enteroids and two independent sample of normal colon were analyzed for transcript expression.

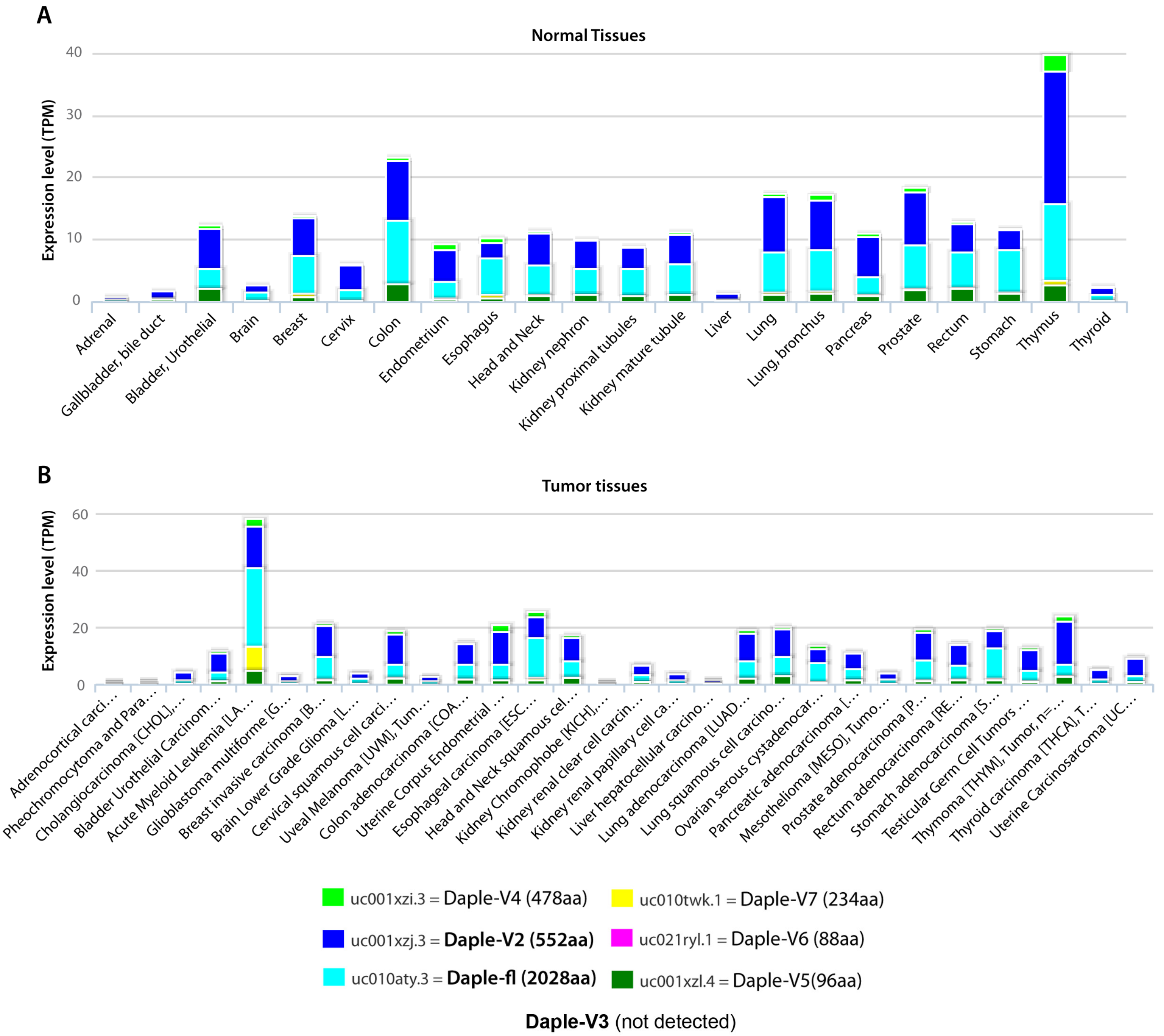

Supplementary Figure 3. Daple-V2 is the predominant isoform expressed across all normal (A) as well as tumor (B) tissues, at levels often similar to that of the RefSeq full length form, Daple-fl. The expression level of various Daple (CCDC88C) isoforms in normal and cancer tissues was analyzed by ISOexpresso, a web-based platform (see *Methods*; http://wiki.tgilab.org/ISOexpresso/). This analysis is performed using the RNA-seq datasets obtained from The Cancer Genome Atlas (TCGA) Data Portal. TPM, Median transcripts per million was used to determine the expression level of isoforms and was calculated by multiplying the median value of the estimates of the transcript in each sample group by one million.

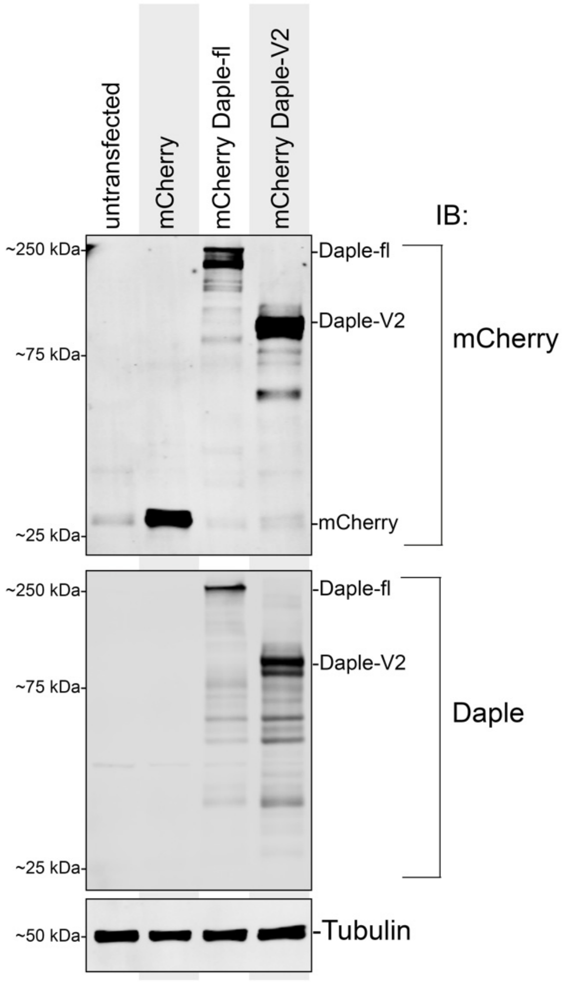

Supplementary Figure 4: Whole cell lysates of HeLa cells transfected with mCherry tagged Daple-fl, Daple-V2, or mCherry tag control. Expression was validated by immunoblotting using antibodies for mCherry or Daple. Tubulin was used for loading control.

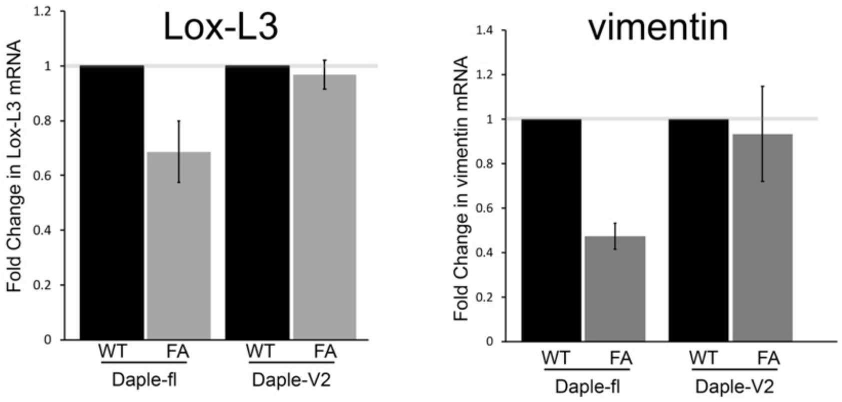

Supplementary Figure 5. mRNA expression of the EMT markers LOX-L3 (*left*) and Vimentin (*right*) were analyzed by qPCR. Results were normalized internally to mRNA levels of the housekeeping gene, GAPDH. Bar graphs display the fold change in each RNA (Y axis) normalized to the expression in cells expressing Daple-fl-WT or Daple-V2-WT. Error bars represent mean ± S.D of 3 independent experiments. The GBA motif of Daple-fl, but not Daple-V2 enhances the expression of genes that trigger EMT. *p* values when comparing WT vs FA: 0.046 (Lox-L3) and 0.021 (Vimentin) for Daple-fl; n.s. for both Lox-L3 and Vimentin in the case of Daple-V2.

A

Cell line	MSI status	CIMP panel 1	CIMP panel 2	CIN	KRAS	BRAF	PIK3CA	PTEN	TP53	Growth suppressive-effect of Aspirin (citation)
DLD-1	MSI	+	+	–	G13D	wt	E545K;D549N	wt	S241F	+++ (this work)
HCT-116	MSI	+	+	–	G13D	wt	H1047R	wt	wt	+++ *
HCT-15	MSI	+	+	–	G13D	wt	E545K;D549N	wt	S241F	+++ *
RKO	MSI	+	+	–	wt	V600E	H1047R	wt	wt	+++ *
SW48	MSI	+	+	–	wt	wt	wt	wt	wt	-/+ *
Caco-2	MSS	+	–	+	wt	wt	wt	wt	E204X	-/+ *
HT-29	MSS	+	+	+	wt	V600E	P449T	wt	R273H	+++ *
SW480	MSS	–	–	+	G12V	wt	wt	wt	R273H;P309S	-/+ *

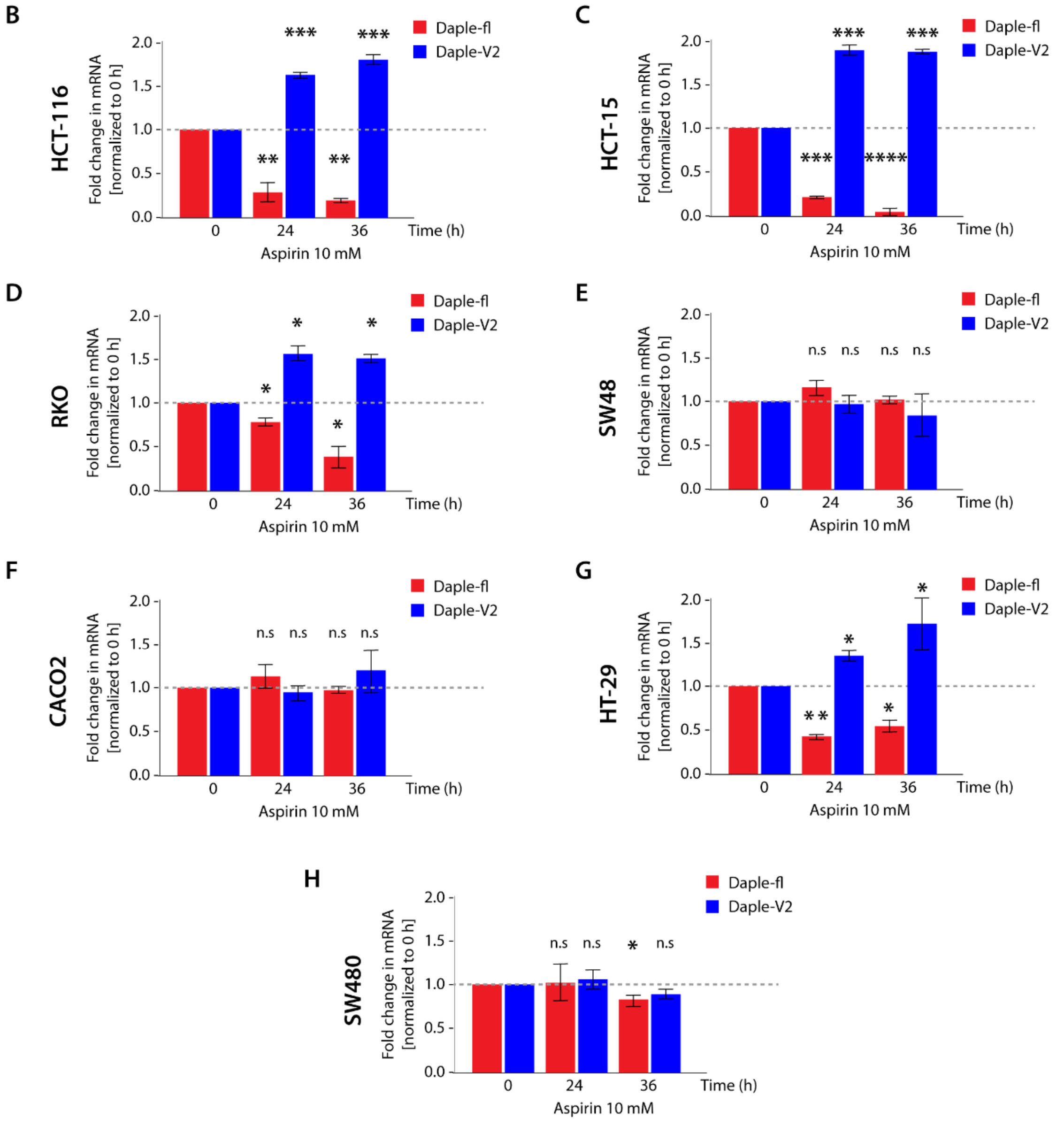

Supplementary Figure 6: **Aspirin increases the ratio of Daple-V2: Daple-fl transcripts in CRC cell lines that harbor PIK3CA mutation.** (**A**) Table listing the CRC cell lines used in studying the impact of Aspirin on the expression of Daple isoforms. *Abbreviations*: CIN, chromosomal instability pathway; MSI, microsatellite instability; MSS, microsatellite stable; CIMP, CpG island methylator phenotype; X, stop codon; fs, frame shift; wt, wild type. Mutations are annotated at the protein level as described by den Dunnen *et al.* [3] and Ahmed *et al.* [4] (standard one-letter amino acid abbreviations). * = Aspirin-sensitivity as documented earlier[5]. (**B-H**) cells were analyzed for Daple-fl and Daple-V2 mRNA by qPCR at indicated time points after exposure to 10 mM Aspirin. Bar graphs display the fold change in each mRNA (Y axis) normalized to the expression levels at 0 hour. Error bars represent mean ± S.D of 3 independent experiments. *p* values: * = <0.05; ** < 0.01; *** < 0.001; **** <0.0001.

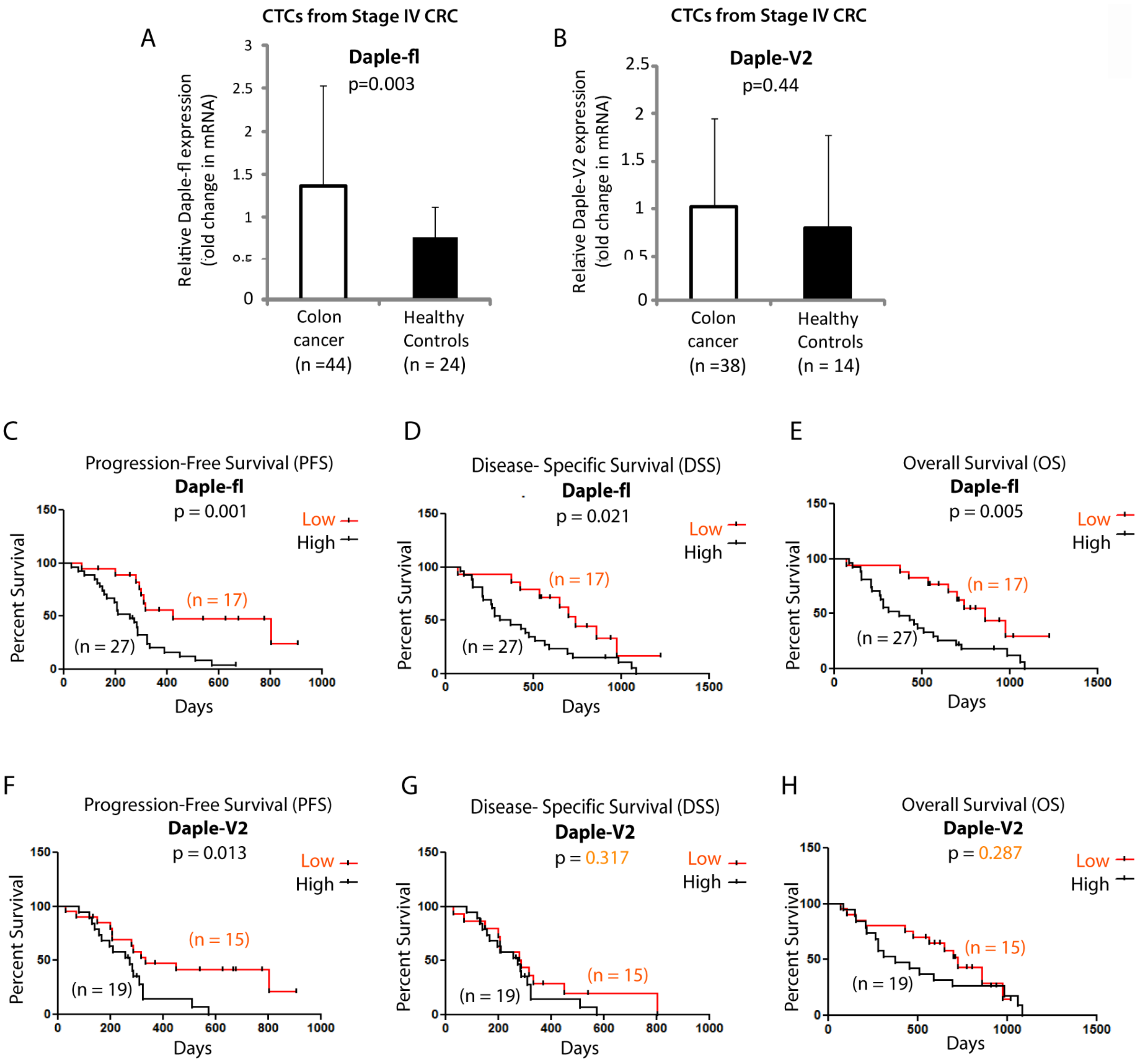

Supplementary Figure 7: **Daple-fl, but not Daple-V2 is elevated in circulating tumor cells (CTCs) and its high expression carries a worse prognosis. (A-B)** EpCAM (epithelial cell adhesion molecule)-immunoisolated CTC fractions from the peripheral blood of patients with metastatic colorectal cancer (stage IV CRC) or from healthy subjects were analyzed for Daple-fl (**A**) or Daple-V2 (**B**) mRNA by qPCR and adjusted for leukocyte contaminants by normalizing to CD45. Bar graph displays the level of Daple expression in each cohort. A normality test confirmed that datasets in both groups were distributed normally. No significant differences were observed in the CD45 levels between two groups (*not shown*). Compared to normal subjects, levels of Daple-fl (**A**), but not Daple-V2 (**B**) mRNA is frequently elevated in CTCs from patients with metastatic colorectal cancer. **(C-H)** Optimal cut-off values for Daple-fl and -V2 mRNA expression were statistically derived (see detailed "*Materials and Methods"*) to generate subgroups of patients with high or low expression levels. Time-dependent progression-free (PFS) and overall (OS) survival probabilities were estimated with the Kaplan-Meier method, and the log-rank test was used to compare the subgroups. Expression of Daple-fl mRNA at high levels in CTCs was associated with poorer progression-free (PFS; **C**), disease specific (DSS; **D**) and overall (OS; **E**) survival in patients with metastatic colorectal carcinoma. Expression of Daple-V2 at high levels in CTCs was associated with poorer PFS (**F**), but had no effect on DSS (**G**) or OS (**H**).

SUPPLEMENTARY REFERENCES

1 Aznar, N. *et al.* Daple is a novel non-receptor GEF required for trimeric G protein activation in Wnt signaling. *Elife* **4**, e07091, doi:10.7554/eLife.07091 (2015).

2 Oshita, A. *et al.* Identification and characterization of a novel Dvl-binding protein that suppresses Wnt signalling pathway. *Genes to cells : devoted to molecular & cellular mechanisms* **8**, 1005-1017 (2003).

3 den Dunnen, J. T. & Antonarakis, S. E. Mutation nomenclature extensions and suggestions to describe complex mutations: a discussion. *Hum Mutat* **15**, 7-12, doi:10.1002/(SICI)1098-1004(200001)15:1<7::AID-HUMU4>3.0.CO;2-N (2000).

4 Ahmed, D. *et al.* Epigenetic and genetic features of 24 colon cancer cell lines. *Oncogenesis* **2**, e71, doi:10.1038/oncsis.2013.35 (2013).

5 Zumwalt, T. J. *et al.* Aspirin-Induced Chemoprevention and Response Kinetics Are Enhanced by PIK3CA Mutations in Colorectal Cancer Cells. *Cancer Prev Res (Phila)* **10**, 208-218, doi:10.1158/1940-6207.CAPR-16-0175 (2017).
